# Comparative genome characterization of the periodontal pathogen *Tannerella forsythia*

**DOI:** 10.1186/s12864-020-6535-y

**Published:** 2020-02-11

**Authors:** Nikolaus F. Zwickl, Nancy Stralis-Pavese, Christina Schäffer, Juliane C. Dohm, Heinz Himmelbauer

**Affiliations:** 1 Department of Biotechnology, Institute of Computational Biology, University of Natural Resources and Life Sciences (BOKU), Vienna, Austria; 2 Department of NanoBiotechnology, NanoGlycobiology unit, University of Natural Resources and Life Sciences (BOKU), Vienna, Austria

**Keywords:** *Tannerella*, Genome assembly, Comparative genomics, Pan-genome, Virulence, Pathogenicity island, Glycosylation gene cluster, Codon usage bias, Computational analysis, Periodontitis

## Abstract

**Background:**

*Tannerella forsythia* is a bacterial pathogen implicated in periodontal disease. Numerous virulence-associated *T. forsythia* genes have been described, however, it is necessary to expand the knowledge on *T. forsythia*’s genome structure and genetic repertoire to further elucidate its role within pathogenesis. *Tannerella* sp. BU063, a putative periodontal health-associated sister taxon and closest known relative to *T. forsythia* is available for comparative analyses. In the past, strain confusion involving the *T. forsythia* reference type strain ATCC 43037 led to discrepancies between results obtained from in silico analyses and wet-lab experimentation.

**Results:**

We generated a substantially improved genome assembly of *T. forsythia* ATCC 43037 covering 99% of the genome in three sequences. Using annotated genomes of ten *Tannerella* strains we established a soft core genome encompassing 2108 genes, based on orthologs present in > = 80% of the strains analysed. We used a set of known and hypothetical virulence factors for comparisons in pathogenic strains and the putative periodontal health-associated isolate *Tannerella* sp. BU063 to identify candidate genes promoting *T. forsythia*’s pathogenesis. Searching for pathogenicity islands we detected 38 candidate regions in the *T. forsythia* genome. Only four of these regions corresponded to previously described pathogenicity islands. While the general protein *O*-glycosylation gene cluster of *T. forsythia* ATCC 43037 has been described previously, genes required for the initiation of glycan synthesis are yet to be discovered. We found six putative glycosylation loci which were only partially conserved in other bacteria. Lastly, we performed a comparative analysis of translational bias in *T. forsythia* and *Tannerella* sp. BU063 and detected highly biased genes.

**Conclusions:**

We provide resources and important information on the genomes of *Tannerella* strains. Comparative analyses enabled us to assess the suitability of *T. forsythia* virulence factors as therapeutic targets and to suggest novel putative virulence factors. Further, we report on gene loci that should be addressed in the context of elucidating *T. forsythia*’s protein *O*-glycosylation pathway. In summary, our work paves the way for further molecular dissection of *T. forsythia* biology in general and virulence of this species in particular.

## Background

*Tannerella forsythia* is a bacterial pathogen associated with human periodontitis, a polymicrobial inflammatory disease of tooth-surrounding tissues [1]. Numerous genes of *T. forsythia* have been reported in the context of the pathogenesis of the disease. Examples include well-described virulence factors such as the leucine-rich-repeat protein BspA [2, 3] and the protease PrtH/Fdf [4]. The *T. forsythia* cell surface (S-) layer was described to consist of the alternating TfsA and TfsB glycoproteins that have their corresponding genes located next to each other in the genome [5–7] and align in a 2D lattice, which drastically impacts the host immune response [8–10]. In *T. forsythia*, the S-layer proteins as well as other cell surface proteins are modified with a complex *O*-glycan that can be dissected in a species-specific portion and a core saccharide that is proposed to be conserved in the *Bacteroidetes* phylum of bacteria [6, 10, 11]. A multi-gene locus encoding the species-specific part of the *T. forsythia* protein *O*-glycan was identified, and the corresponding protein *O*-glycosylation pathway has been recently explored in detail [10]. Following assembly of the glycoprotein in the bacterial periplasm, the S-layer glycoproteins are targeted via their conserved C-terminal domain (CTD) to a type IX secretion system (T9SS) for export across the outer membrane [12]. The T9SS is a recently discovered, complex translocon found only in some species of the *Bacteroidetes* phylum [13], and CTDs, typically consisting of 40–70 amino acids and sharing an immunoglobulin-superfamily (IgSF) domain, are present in many other proteins in *T. forsythia*. The glycobiology repertoire of the *T. forsythia* genome also contains numerous glycosidases and carbohydrate-active enzymes that require attention within the context of virulence [14]. Further, a sialic acid utilization gene locus encoding a transporter and involved enzymes have been shown to play an important role for the species to thrive within the oral biofilm community [15–17]. Apart from the capability of cleaving oligosaccharides, the niche and suggested role in pathogenesis requires the species to produce proteolytic enzymes; in addition to PrtH, much attention has been directed to a set of six proteases of similar protein architecture which contain a modified CTD, terminating with the amino acid sequence KLIKK, hence termed KLIKK proteases [18]. Whereas the roles of these and other suggested virulence factors continue to be explored, the search for novel virulence factors may be required to complete the picture on *T. forsythia’s* contributions and role in pathogenesis.

Previous characterizations of the *T. forsythia* virulence factors were mostly based on the American Type Culture Collection (ATCC) 43037 type strain employing wet-lab experimentation, whereas computational analyses of the virulence-related gene repertoire mostly used the genome sequence of strain FDC 92A2. Although FDC 92A2 was the first fully sequenced *T. forsythia* strain available [19], the resulting genome assembly was incorrectly labelled and deposited as ATCC 43037 in the National Center for Biotechnology Information (NCBI) databases. This discrepancy was not noticed by the research community until many years later. Because of inconsistent results and sequence mismatches, initially interpreted as sequencing errors or as misassemblies in the genomic reference, *T. forsythia* was sequenced again and a genuine genome assembly for ATCC 43037 was generated [20]. Meanwhile, the strain attribution error has been corrected in the NCBI databases, but persists in other databases.

The *T. forsythia* ATCC 43037 genome assembly published by Friedrich et al. was a draft genome assembly, consisting of 141 contigs with an N50 contig length of 110 kbp. Even though this has substantially improved the genomics resources available for *T. forsythia*, a more contiguous and more complete genome assembly is required for many analyses, especially for whole-genome comparative approaches. Furthermore, the genome assembly of strain FDC 92A2 remained in the NCBI databases as reference genome for *T. forsythia* due to its completeness. However, the cultivation of FDC 92A2 has been reported to be unreliable [21], so that ATCC 43037 will certainly continue to be the most widely used strain in research labs. In addition to the genome assemblies of ATCC 43037 and FDC 92A2, genome assemblies of eight further *T. forsythia* strains have become available in recent years [22–25].

Within the genus *Tannerella, T. forsythia* is the only well characterized species. Several isolates from various origins have been assigned to the genus *Tannerella* [26]; until recently, however, none of these have been successfully cultivated, hampering their characterization.

*Tannerella* sp. BU063 (also referred to as Human Microbial Taxon ID 286 or HMT 286) is of special interest, as it is considered a putative periodontal health-associated strain. Following recent successful cultivation [27], a complete and gap-free genome assembly of *Tannerella* sp. BU063 has become available replacing a previously generated highly fragmented assembly [28].

Overall, the currently available genomes from the genus *Tannerella* enable comparative genomics approaches to (i) continue searching for novel *T. forsythia* virulence factors, (ii) confirm the relevance of previously reported or suggested virulence factors throughout the *T. forsythia* species, and (iii) explore features of the *T. forsythia* genome that might be of interest beyond the organism’s virulence.

Here, we present a new, more contiguous genome assembly for the *T. forsythia* ATCC 43037 type strain, which is based on sequences of the published draft assembly and, hence, is compatible with previous studies and gene annotations. Further, we use this improved genome assembly together with genome assemblies from nine additional *T. forsythia* isolates and from the putative health-associated relative *Tannerella* sp. BU063 in comparative genomics approaches.

## Results

### Improved assembly of the Tannerella forsythia type strain ATCC 43037

The genome of the *T. forsythia* ATCC 43037 type strain had been assembled previously [20] based on Illumina paired-end sequencing data resulting in an assembly of 141 contigs with an N50 size of 114 kilobasepairs (kbp) (Table 1). The largest sequence was 487 kbp comprising about 15% of the total assembly size of 3.282 Megabasepairs (Mbp). In order to improve the contiguity of the assembly, we generated a new data set of 11 million Illumina mate-pairs with read length of 2 × 125 nucleotides (nt), corresponding to 800-fold genome coverage, and showing a peak span size of 1.8 kbp (Additional file 10: Figure S1). We used both the published paired-end sequencing reads downsampled to a coverage of 100-fold and the newly generated mate-pairs to build connections between the contigs of the ATCC 43037 genome assembly generated by Friedrich et al. [20]. After scaffolding and gap filling, the N50 length increased to 1.85 Mbp and the number of sequences decreased to 87. The total assembly size increased slightly to 3.296 Mbp due to gaps between contigs. The three largest sequences (1.85 Mbp, 859 kbp, 532 kbp) encompassed 99.1% of the assembly. The fraction of undetermined bases within scaffolds was very small (0.26%). Thus, the new assembly of strain ATCC 43037 can be considered as essentially complete. The genome sizes of three fully sequenced *T. forsythia* strains were slightly larger, namely 3.40 Mbp (FDC 92A2) [19], 3.39 Mbp (KS16), and 3.35 Mbp (3313) [22], respectively, with an average genome size of 3.38 Mbp. Taking this average genome size as a basis the average gap size in the new ATCC 43037 assembly was 900 bp between scaffolds.

**Table 1:** *Tannerella* genome assemblies analysed including the ATCC 43037 assembly generated in this work

Strain name	GenBank Accession	Genome size [bp]	# of sequences	% GC	RefSeq Annotation Date
*Tannerella forsythia*					
ATCC 43037	VFJI00000000 (this work)	3,296,274	87	47.1	–
ATCC 43037	JUET00000000.1	3,281,748	141	47.1	06/12/2017
FDC 92A2	NC_016610.1	3,405,521	1	47.0	10/21/2017
3313	NZ_AP013044.1	3,350,939	1	47.1	04/04/2017
KS16	NZ_AP013045.1	3,393,002	1	47.2	04/04/2017
UB4	FMMN00000000.1	3,233,032	71	47.2	06/12/2017
UB22	FMML00000000.1	3,272,368	98	47.1	06/12/2017
UB20	FMMM00000000.1	3,252,894	93	47.1	06/12/2017
9610	MEHX00000000.1	3,201,941	79	47.3	06/12/2017
W11663	NSLJ00000000.1	3,300,179	140	47.1	10/14/2017
W10960	NSLK00000000.1	3,312,685	98	47.2	10/14/2017
*Tannerella* sp. BU063					
n/a	CP017038.1	2,973,531	1	56.5	04/13/2017

**Fig. 1:**
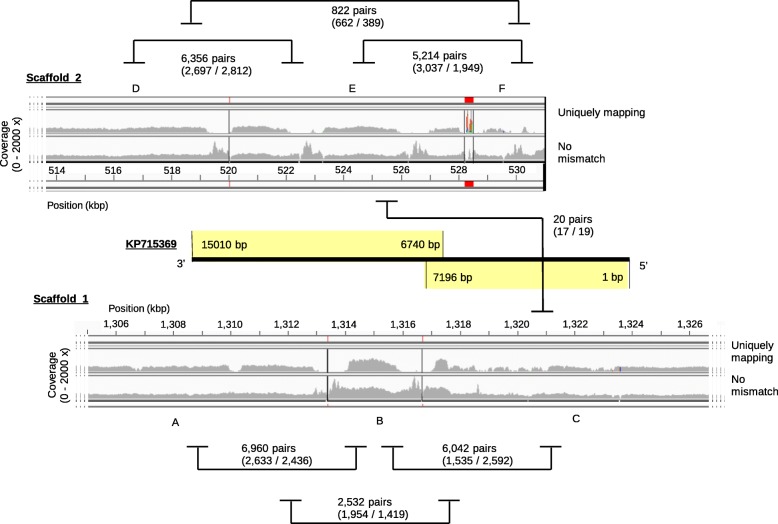
Comparison of our assembled scaffolds to a previously published *T. forsythia* sequence. The sequence KP715369 (black bar in the middle) aligns partially to our scaffold 1 (bottom) and partially to scaffold 2 (top). The sections named A to F represent the scaffolded contigs, gaps between them are indicated by vertical bars. Coverage tracks are shown for two different mapping strategies (allowing zero mismatches versus allowing only uniquely mapping reads); the differences between the two tracks highlight repetitive content found especially at the contig ends. Numbers of linking read pairs between contigs are indicated (based on the uniquely-mapping strategy) along with the numbers of unique mapping positions (read 1 / read 2). There were only 20 read pairs that supported the linkage of contig C to contig E as suggested by the alignment of KP715369. All adjacent contigs as scaffolded by us were supported by more than 5000 pairs for each link

We compared our ATCC 43037 assembly to a published 15 kbp-long genomic sequence (GenBank accession KP715369) of the same *T. forsythia* strain [18] resulting in a conflicting alignment. About one half of the sequence published by Ksiazek et al. aligned to a non-terminal region in scaffold 1 and the other half aligned to a non-terminal region in scaffold 2 in our assembly. We carefully checked the sequencing reads that supported our connections and also mapped our reads to the 15-kbp sequence. Reduced read coverage was found in all breakpoint regions, but several thousands of connecting mate-pairs supported our version compared to only twenty mate-pairs that would confirm the continuity of the 15-kbp sequence (Fig. 1). When comparing the 15-kbp sequence to the published genome assemblies of *T. forsythia* strains 92A2, 3313, and KS16, we did not find the 15-kb sequence to align continuously in any of these strains, however, the majority of the produced alignments were found within single regions of each of the three genomes. While some parts of the 15-kbp sequence aligned also to other regions, a distinct split, as described above for ATCC 43037, could not be observed (Additional file 12: File S1). We note that Ksiazek et al. published their work at a time when it was not yet clear that the *T. forsythia* reference genome attributed to ATCC 43037 was in fact derived from strain 92A2 [20]. Hence, Ksiazek et al. may have unknowingly relied on strain 92A2 instead of ATCC 43037 for guiding their sequencing and assembly strategy.

### Comparative analysis of Tannerella sp. genome assemblies

**Fig. 2:**
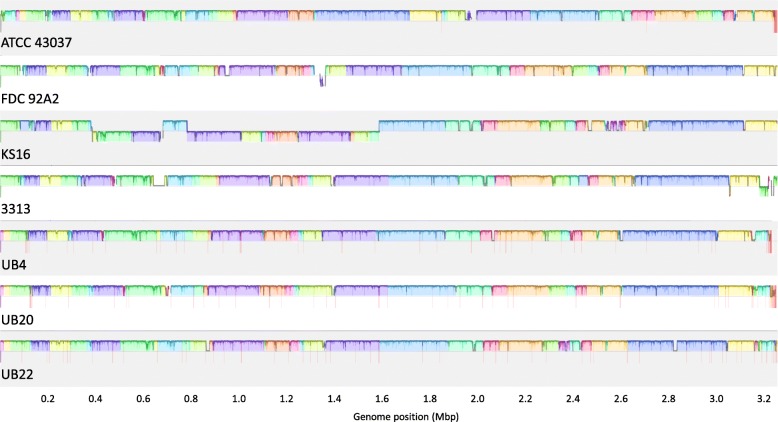
Multiple whole genome alignment of eight *T. forsythia* strains. Each coloured block represents a genomic region that aligned to a region in at least one other genome, plotted in the same colour, to which it was predicted to be homologous based on sequence similarity. Blocks above the centre line indicate forward orientation; blocks below the line indicate reverse orientation relative to strain 92A2. A histogram within each block shows the average similarity of a region to its counterparts in the other genomes. Red vertical lines indicate contig boundaries. Strain ATCC 43037 displayed two translocations compared to strain 92A2 with lengths of approximately 500 kbp (blue and yellow blocks at the right end of 92A2 and in the centre of ATCC) and 30 kbp (pink block at approx. 1.25 Mbp in 92A2 and at approx. 2.7 Mbp in ATCC), respectively. Previously described large-scale inversions in strain KS16 could be confirmed (reverted blocks in the left half of the alignment)

Our new genome sequence allowed whole-genome comparisons with other *Tannerella* assemblies to assess genomic structural differences and gene order conservation. We compared the available genome assemblies of six disease-associated *T. forsythia* strains - 92A2, 3313, KS16, UB4, UB20, and UB22 - with the assembly of strain ATCC 43037, together with the putative health-associated *Tannerella* sp. BU063 isolate in whole-genome alignments (Table 1). Genome assemblies of a close relative of *Tannerella* sp. BU063 dubbed *Tannerella* sp. BU045 were recently released [29] based on data that were acquired by single-cell sequencing. Considering the degree of assembly fragmentation (about 600 contigs, N50 of about 22 kbp), data derived from this isolate were not used for the current work. We chose strain 92A2 as a reference because of its completeness and aligned the other strains against it. The alignments revealed that all *T. forsythia* strains shared highly conserved genome structures (Fig. 2). Three of the assemblies showed considerable fragmentation (strain UB4: 71 contigs, UB20: 93 contigs, UB22: 98 contigs) so that large-scale rearrangements could not be analysed. However, 78–83% of the assembled contigs per strain aligned to strain 92A2 with at least 80% of their length and minimal sequence identity of 80%, taking alignments with a minimum length of 250 bp into account. Only a few contigs that could not be aligned to the 92A2 reference under these conditions exceeded 1000 bp (one, six, and seven contigs for UB4, UB20, and UB22, respectively), comprising only 2–8% of the total assembly lengths (Table 2). Reducing the required alignment length from 80 to 50%, more than 99.5% of each assembly aligned to the 92A2 reference. Similarity blocks as detected throughout all compared strains spanned contig boundaries in many cases suggesting a high degree of collinearity even between the fragmented assemblies.

**Table 2:** Alignable fraction of nine *T. forsythia* strains and *Tannerella* sp. BU063 in whole-genome alignments against *T. forsythia* strain FDC 92A2 as reference sequence. Results are based on blastn output. The scaffolded ATCC 43037 assembly generated in this work was used

Strain name					
	> = 99% seq identity	> = 95% seq identity	> = 80% seq identity	> = 70% seq identity	> = 50% seq identity
*Tannerella forsythia*					
ATCC 43037	40.58	88.52	91.46	92.15	92.59
3313	44.27	87.68	92.00	92.56	92.76
KS16	43.43	90.63	92.72	93.24	93.55
UB4	42.61	88.47	92.59	93.14	93.29
UB22	51.94	90.99	92.02	93.01	93.36
UB20	49.89	90.54	93.30	93.68	93.89
9610	42.58	87.86	90.35	90.87	91.21
W11663	47.50	90.30	92.50	92.94	93.06
W10960	44.83	88.75	91.70	92.55	92.92
average	45.29	89.30	92.07	92.68	92.96
*Tannerella* sp. BU063					
	> = 95% seq identity	> = 80% seq identity	> = 70% seq identity	> = 50% seq identity	> = 30% seq identity
n/a	0.00	0.97	24.38	38.25	38.37

The genomes of strains 92A2, 3313 and KS16 had been assembled into one contiguous sequence, and, thus, were most informative regarding potential rearrangements within the *T. forsythia* species. The alignments confirmed two large inversions in strain KS16 when compared to 92A2 or 3313, and a high degree of collinearity between the latter two, as reported previously [22]. Our ATCC 43037 assembly was found to show two large-scale rearrangements when compared to strains 92A2 and 3313, respectively. One of these rearrangements disrupted the larger of the two KLIKK protease loci, which was contained within the 15-kbp sequence mentioned above.

**Fig. 3:**
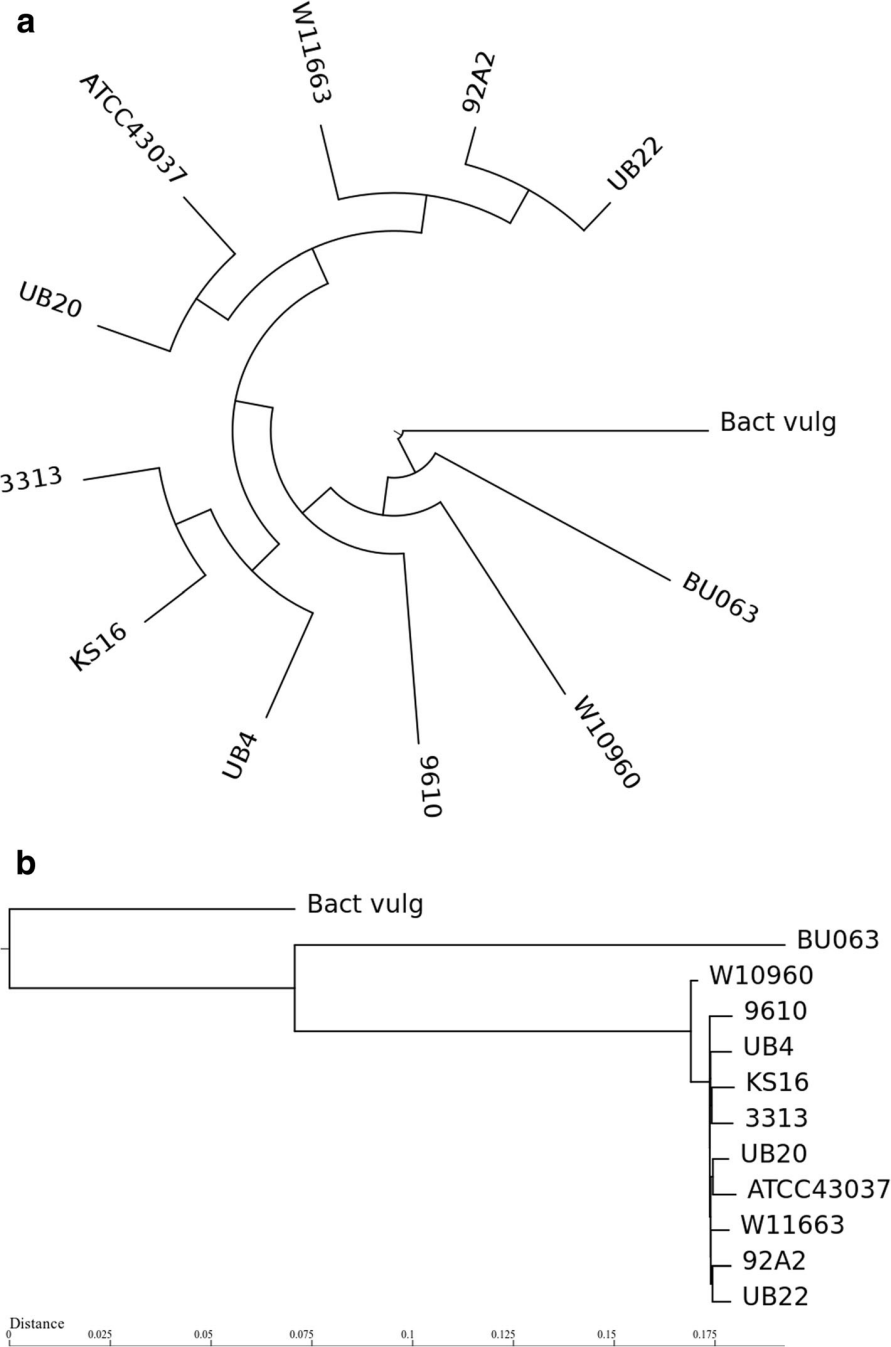
Phylogenetic tree showing the topology (**a**) and the distances (**b**) as computed by MASH applied on the whole-genome assemblies of *T. forsythia* strains and *Tannerella* sp. BU063, including *Bacterioides vulgatus* ATCC 8482 as outgroup

In order to investigate the relatedness among the 10 *T. forsythia* strains and *Tannerella* sp. BU063, we performed a phylogenetic analysis. We determined pairwise distances between the assembled genomes using Mash [30] and included *Bacteroides vulgatus* ATCC 8482 as an outgroup. The resulting distance matrix was used to calculate a phylogentic tree using the Fitch-Margoliash algorithm. The phylogenetic tree clustered the ten *T. forsythia* isolates closely together and showed *Tannerella* sp. BU063 as a separate sister taxon. The distance of *T*. sp. BU063 to the *T. forsythia* subtree was almost as large as the distance of the outgroup (Fig. 3 a, b).

We found large differences to the genome structure of the putative periodontal health-associated isolate *Tannerella* sp. BU063. When aligning the genome assemblies of nine disease-associated strains - ATCC 43037, 3313, KS16, UB4, UB20, UB22, 9610, WW11663, and WW10960 - to the genome of strain 92A2, on average 92.1% of the 92A2 sequence was covered (match length cut-off 250 bp; minimum sequence identity 80%), and 41 to 52% were found to be covered even when raising the sequence identity threshold to 99%. In contrast, the genome sequences of the putative periodontal health-associated phylotype *Tannerella* sp. BU063 covered less than 1% of the 92A2 genome by alignments with a sequence identity of at least 80%. Even when lowering the sequence identity cut-off to 70 and 50% the alignments covered only 24 and 38% of the 92A2 sequence, respectively.

**Fig. 4:**
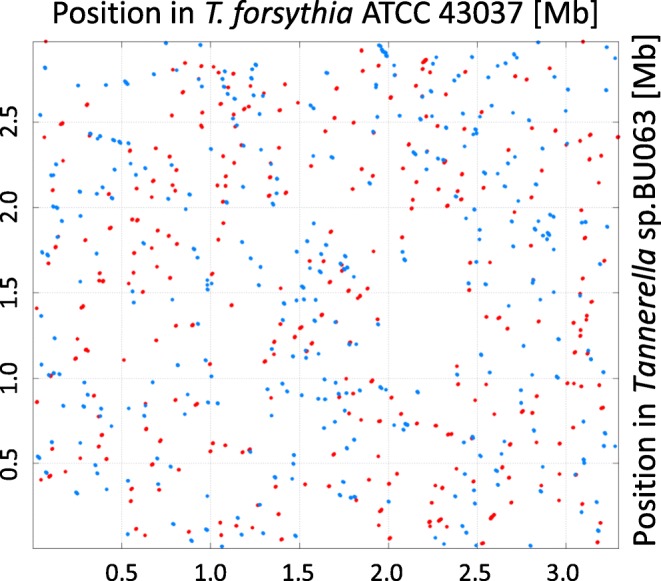
Whole genome alignment between the six frame amino acid translations of both *Tannerella* sp. BU063 and the scaffolded and ordered ATCC 43037 assembly. Whereas the amino acid alignment reflects similarity with respect to gene content, the order of genes is not preserved

Similarly, our findings confirmed that the gene order in *T. forsythia* compared to *Tannerella* sp. BU063 was largely changed. Loss of synteny had been reported previously based on highly fragmented genome assemblies [28]. Here, we used the complete and gap-free genome sequence of *Tannerella* sp. BU063 (Table 1) enabling genome-wide analysis beyond previous breakpoints. Although 55% of the genes encoded within the *Tannerella* sp. BU063 genome were found to have an ortholog in at least six different *T. forsythia* strains, our genomic alignment indicated that the gene order was shuffled (Fig. 4).

In each of the assemblies of 3313, 92A2, and ATCC 43037 we found one continuous sequence of at least 20 kbp that indicated a strain-specific region to which no other strain contained a homologous segment that could be aligned well. The strains KS16 and 3313, both of them isolated from periodontitis patients in Japan, shared a homologous block that was specific to these two strains which encompassed a gene annotated as a transposase, surrounded by numerous genes that had been annotated as hypothetical proteins of unknown function [22]. We expect further strain-specific regions of similar size as well as strain-specific genes in the other genomes. The individual location of strain-specific regions in 3313, 92A2, ATCC 43037 suggested that such regions occur dispersed throughout the genomes.

In summary, these results and the alignments shown in Fig. 2 illustrate the high degree of conservation with respect to sequence content as well as genome structure throughout the *T. forsythia* species and provide genomic evidence to suggest the re-classification of *Tannerella* sp. BU063 as a separate species.

### Comparative assessment of Tannerella virulence factors

Currently available *T. forsythia* genomes contain 2600–2700 protein-coding genes, many of which lack functional annotation. The increasing wealth of knowledge contained in sequence databases may provide functional predictions for these genes in the future. At present, however, we may reveal candidate genes involved in pathogenesis by comparing complete genomes from strains of known pathogenic and non-pathogenic nature, even if their genes are not yet functionally annotated. Such an approach is especially interesting in the case of *T. forsythia*, as its cultivation requirements make a systematic knock-out approach very challenging.

**Fig. 5:**
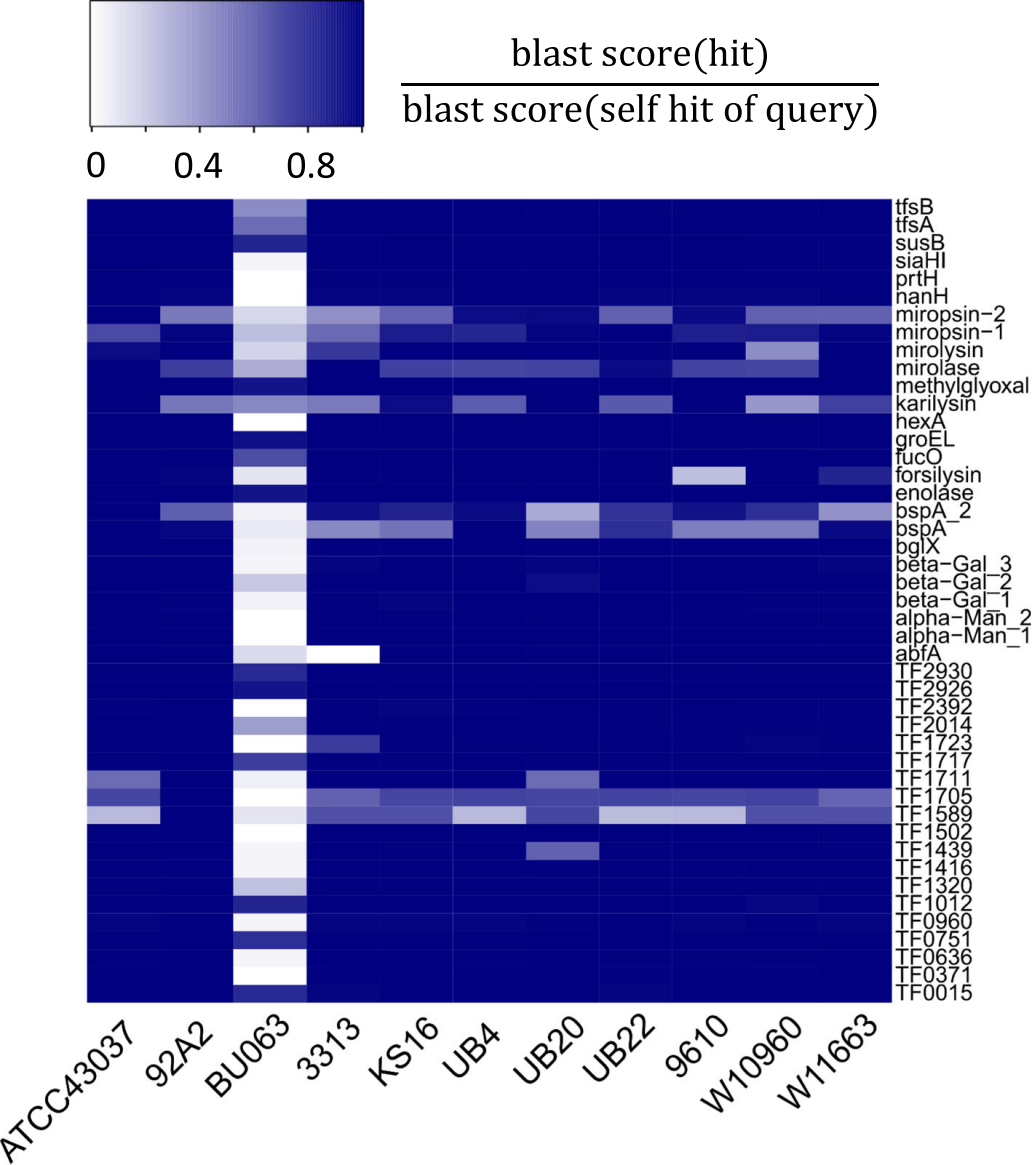
Blast Score Ratio (BSR) values plotted as heatmap for 45 suggested virulence genes in ten *T. forsythia* strains and the genome of putative health-associated *Tannerella* sp. BU063. Gene sequences were blasted against the complete genomic sequences of each genome. *Tannerella* sp. BU063 achieved considerable BSR values for several genes that were actually suggested as virulence factors in pathogenic *T. forsythia* strains. On the other hand, some of the pathogenic strains show reduced similarity to some predicted virulence factors

A number of genes have so far been suggested to be associated with the pathogenicity of *T. forsythia* [18, 31–33]. We assessed the presence or absence of functional orthologs of such genes within genome assemblies of ten different *T. forsythia* strains, as well as within the putative periodontal health-associated genome of *Tannerella* sp. BU063. We employed BLAST score ratio (BSR) values for the gene comparisons as calculated with LS-BSR [34], whereby the blast score of the alignment of two genes that match each other is normalized by dividing the result by the blast score obtainable in a self-hit of the query. This yields a value of 1 for identical sequences and a value of zero for sequences which are entirely unrelated. We included 45 potential virulence-related genes and determined their BSR values in all eleven strains by applying LS-BSR on the entire genomes (Fig. 5, Additional file 1: Table S1) and on the annotated gene sets (Additional file 11: Figure S2, Additional file 2: Table S2). High BSR values suggest that a functional ortholog to a pathogenicity-associated gene is present in a certain strain, while BSR values < 0.4 indicate likely absence of a functional ortholog of this gene [34]. The two input data sets resulted in comparable BSR values for most genes. Differences in BSR values (differing by 0.2 or more: TfsA in one strain, mirolysin in one strain, karilysin in two strains, and TF2392 in three strains) might indicate incorrectly annotated genes in particular strains or truncated gene sequences due to mutations of start or stop codons.

Based on the comparison of entire genomes our result showed generally high BSR values for virulence factors in the pathogenic *T. forsythia* strains and low BSR values in *Tannerella* sp. BU063 (Fig. 5, Additional file 1: Table S1). However, BSR values > = 0.7 indicated 11 pathogenicity-associated genes as present in *Tannerella* sp. BU063 (of which four genes had BSR > = 0.9: methylglyoxal synthase, GroEL, enolase, TF2925). Four genes with BSR < 0.4 indicated absence in at least one of the pathogenic strains (forsilysin in strain 9610; BspA_2 in UB20; AbfA in 3313; TF1589 in ATCC 43037, UB4, UB22, and 9610) (Additional file 1: Table S1) providing evidence that re-evaluation of the virulence and other phenotypical characteristics of strains 9610, UB20, 3313 may be required and that TF1589 may be of lower or no importance for the pathogenicity of *T. forsythia*.

Varying BSR values indicated sequence variation between different *T. forsythia* strains for the surface antigen BspA*,* one of the most comprehensively described virulence factors of *T. forsythia* and linked to pathogenesis by in vivo studies [2, 3]. As noted previously [31], there were six putative BspA homologs predicted in the genome of strain 92A2 besides the main BspA gene. For the gene most similar to the latter, termed BspA2, notable sequence variation was indicated as well. Both BspA and BspA2 showed BSR values close to zero (0.09 and 0.06, respectively) in *Tannerella* sp. BU063 indicating their absence*.* Variation was also found within the group of KLIKK proteases (i.e. miropsin-1, miropsin-2, mirolysin, mirolase, karilysin, forsilysin). In concordance with a previous study that described high-identity KLIKK protease homologs to be absent from *Tannerella* sp. BU063 but found a truncated mirolase-like open reading frame [18], we detected a homolog of mirolase with 46% sequence identity at 86% sequence coverage in the *Tannerella* sp. BU063 genome included here. As it was the best bidirectional hit, it is likely that it represents a true mirolase ortholog. The corresponding gene (NCBI protein database accession WP_069175679.1) is almost identical (97% identity at 99% coverage) with the gene reported by Ksiazek et al. which was described to share a high degree of similarity in the catalytic domain with KLIKK proteases, but lacks a signal peptide, lacks an N-terminal pro-fragment, and lacks the variable region that is characteristic of the C-terminal extension in KLIKK proteases.

In contrast to previous reports [28], we found a best bidirectional hit for karilysin in the *Tannerella* sp. BU063 assembly, in this case with 53% identity over the entire length of the gene. Within the work of Beall et al., only homology to the C-terminal part containing the secretion signal domain was reported. Interestingly, a large fraction of the dissimilarity between *T. forsythia* karilysin, as described by Ksiazek et al. [18], and the putative ortholog (NCBI protein database accession WP_083206853) identified in the gap-free *Tannerella* sp. BU063 genome assembly was found in regions other than the catalytic domain of the protein (Additional file 13: File S2).

Further work will be required to determine whether this gene is a *bona fide* functional karilysin ortholog. In any case, the previous conclusion that KLIKK proteases are completely absent from the *Tannerella* sp. BU063 genome has to be revised.

As potential targets for therapeutic strategies we would consider only those genes that are highly conserved in all *T. forsythia* strains, but absent or weakly conserved in the putative health-associated *Tannerella* sp. BU063 isolate, e.g. out of the 45 potential virulence factors the 20 genes showing a BSR of 0.9 or larger in *T. forsythia* strains and a BSR smaller than 0.6 in *Tannerella* sp. BU063 (Additional file 3: Table S3).

### Analysis of the T. forsythia pan-genome and comparison to Tannerella sp. BU063

**Fig. 6:**
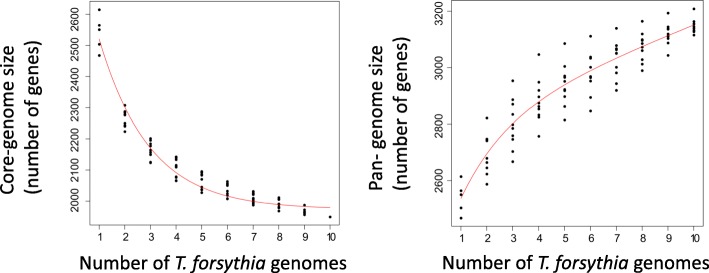
Predicted core- and pan-genome sizes for *T. forsythia* based on ten genome assemblies using a sampling approach that iteratively adds genomes to the analysis. The species’ core genome has a saturated size of 1900 genes, i.e. genes that are found to be conserved throughout the ten analysed strains are likely to be conserved throughout the whole species (left panel). In contrast, novel genes are expected to be found in newly sequenced *T. forsythia* genomes as indicated by the pan-genome curve that has not yet reached a saturation plateau (right panel)

The comparison of gene repertoires encoded within different genomes of the same species has indicated remarkable flexibility [35, 36]. For a particular species, a certain set of genes will be found in all of the studied genomes, while some genes will be restricted to just a subset thereof. The former genes will be referred to as the core genome, while the overall gene composition encompassing genes which may be present in just a single accession is called the pan-genome. Frequently, an extended version of the core genome is determined as well: As soon as genome comparisons take advantage of draft assemblies which may encompass gaps, the chance of finding additional core genes increases. Therefore, criteria are specified which demand core genes to be present in at least 80% or 90% of the studied genomes, respectively. Genes which meet such thresholds are assigned to a soft core genome. Based on the currently available annotated genomes of ten different strains of *T. forsythia*, i.e. ATCC 43037, 3313, KS16, UB4, UB20, UB22, 9610, WW11663, WW10960, and 92A2, we assessed a core genome of the species comprising 1864 genes, when requiring a core gene to be present in each strain without exception. Using less stringent criteria, further genes could be assigned as core genes. A soft core genome which required a gene to be present in > = 90% of the strains contained 2043 genes; reducing the required threshold to > = 80%, the number of genes increased to 2108. Analysis of the number of genes after iterative addition of the ten strains revealed saturation of the gene number in the core genome, whereas the pan genome of the species may still increase when analysing more strains (Fig. 6).

Of the genes found in the *T. forsythia* soft core genome (detection in > = 80% of strains), 894 were found to not possess an ortholog in the putative periodontal health-associated species *Tannerella* sp. BU063, hence, these genes will likely encompass genes that are associated with pathogenicity. Searching for Kyoto Encyclopedia of Genes and Genomes (KEGG) orthology terms overrepresented in this gene set turned out to be inconclusive, because such terms had been assigned to only about a quarter of the genes. We therefore adopted a different strategy to identify new candidate loci involved in the virulence of *T. forsythia* (see below).

### Novel putative virulence factors and pathogenicity islands

Genes involved in pathogenesis often co-localize in bacterial genomes within pathogenicity islands. Some strategies that aim at the identification of pathogenicity islands or other genomic islands make use of described structural elements that are common to such islands [37]. Here, we employed a comparative approach with respect to the *Tannerella* sp. BU063 genome to identify putative pathogenicity islands in *T. forsythia* strain 92A2. We defined a putative pathogenicity island as a genomic region in *T. forsythia* strain 92A2 that contained at least five consecutive genes, (i) which were part of the *T. forsythia* soft-core genome (detection in > = 80% of strains) and (ii) which could not be found in the *Tannerella* sp. BU063 genome.

In total, we detected 38 such regions, of which 18 encompassed at least seven consecutively ordered genes (Additional file 9: Table S9). Five of the island candidates contained more than ten such genes. Three of these regions are known from an initial genomic comparison between *T. forsythia* and *Tannerella* sp. BU063 [28], one further region corresponds to the sialic acid utilization locus [15]. Notably, nine of the islands encode SusD/TonB/TolC-like components indicative of polysaccharide utilization loci (PULs). PULs are a unique feature of *Bacteroidetes* genomes encoding physically-linked carbohydrate-active enzymes next to an outer membrane transporter and a carbohydrate sensor/transcriptional activator and are important for colonization of nutritional niches [38, 39].

Three islands reported previously [28] were not detected by our approach. This discrepancy likely results from methodological differences: First, Beall et al. analysed a fragmented *Tannerella* sp. BU063 genome assembly derived from a single-cell genomics approach, whereas we took advantage of a gap-free genome assembly of *Tannerella* sp. BU063 generated after successful in vitro cultivation of this species. Second, in the previous reports all genes from *T. forsythia* strain 92A2 (misclassified as ATCC 43037) were considered, whereas we focused on genes of the *T. forsythia* soft core genome. Hence, the results by Beall et al. may include gene clusters derived from the 92A2 accessory genome (25% of genes of a *T. forsythia* strain) and might not be representative for the species as a whole.

### Protein O-glycosylation pathway genes

The general protein *O*-glycosylation gene cluster of *T. forsythia* was described to contain a number of glycosyltransferases (Gtfs) and other enzymes involved in the biosynthesis of *Tannerella*’s *O*-glycan structures [10]. However, the genes required for the initiation of the glycan synthesis have yet to be determined. Based on demonstrated analogies in the biosynthesis pathways of different bacterial glycoconjugates [40] it is conceivable that an initiating WbaP-like enzyme, like acting in the *O*-antigen biosynthesis of *Salmonella enterica* [41], is also involved in *O*-glycan biosynthesis of *T. forsythia*. WbaP transfers galactose to an undecaprenyl-phosphate carrier residing within the inner membrane with the phosphate group facing the interior. Further sugar moieties are added to the nascent glycan structure from activated sugar precursors before it is flipped across the membrane by the flippase Wzx. Two such candidate genes were found in each *T. forsythia* strain included in this work. For the ATCC 43037 strain these were Tanf_04030 (WP_046824981.1), annotated as a glycosyltransferase, and Tanf_09660 (WP_014226155.1), annotated as an undecaprenyl-phosphate glucose phosphotransferase. Both of these candidate genes had previously been knocked out individually, but no effect on *O*-glycan synthesis was observed (Gerald Posch, Bettina Janesch, and Christina Schäffer, unpublished data). There are numerous predicted Gtfs in the *T. forsythia* genome that are yet uncharacterized. Knock-out experiments for all of them would present a possible approach to further elucidate *O*-glycan biosynthesis, however, due to *T. forsythia*’s slow growth and fastidious growth requirements, a rather tedious one. While it cannot be ruled out that the missing glycosylation pathway components are encoded by genes dispersed throughout the genome, it may be speculated that they are also co-located in a certain region. We searched for such putative glycosylation loci in the complete genome assembly of strain 92A2. In brief, a putative glycosylation locus was defined as an interval containing at least three predicted Gtfs or genes containing Gtf-associated domains within a stretch of 15 consecutive genes. Six such regions encompassing putative glycosylation loci could be discovered (Table 3), and one of them was found to be partially conserved in two different *Parabacteroides* genomes. Two regions, each, were found to be partially conserved in a single species, one in *Bacteroides fragilis* and the other one in *Tannerella* sp. BU063. These loci represent suitable starting points for further experimentation in order to confirm their role in glycosylation.

**Table 3:** Positions of putative glycosylation (PGL) loci in *T. forsythia* strain FDC 92A2

Locus tag (RefSeq, GenBank)	Position	Strand	Protein ID, Description	Conserved Domains	dbCAN
PGL_1					
BFO_RS00485, BFO_0104	119,936–121,180	+	WP_014223582.1, glycosyltransferase group 1 family protein	cl28208 RfaB superfamily	GT4
BFO_RS00535, BFO_0114	132,395–133,165	+	WP_014223590.1, hypothetical protein	cl11394 Glyco_tranf_GTA_type superfamily	–
BFO_RS00545, BFO_0116	133,715–134,380	+	WP_041590509.1, hypothetical protein	cl11394 Glyco_tranf_GTA_type superfamily	–
BFO_RS00550, BFO_0117	134,417–135,097	+	WP_014223593.1, glycosyl transferase	cl01298 Glyco_transf_25 superfamily	GT25
PGL_2					
BFO_RS02100, BFO_0467	500,734–501,384	–	WP_052299218.1, hypothetical protein	cl02988 Glyco_transf_10 superfamily	GT10
BFO_RS02105, BFO_0468	502,333–504,648	–	WP_014223924.1 penicillin-binding protein 1C	TIGR02073 PBP_1c	GT51
BFO_RS02135, BFO_0475	513,630–514,787	+	WP_014223931.1 mannosyltransferase	cd03809 GT1_mtfB_like	GT4
PGL_3					
BFO_RS02420, BFO_0544	586,421–587,608	+	WP_014223997.1, glycosyl transferase family 1	cl10013 Glycosyltransferase_GTB_type	GT4
BFO_RS02430, BFO_0547	588,656–589,774	+	WP_014223999.1, glycosyl transferase family 1	cl10013 Glycosyltransferase_GTB_type	GT4
BFO_RS02435, BFO_0564	589,763–590,959	–	WP_014224000.1, hypothetical protein	cl10013 Glycosyltransferase_GTB_type	–
PGL_4					
BFO_RS07405, BFO_1699	1,808,692–1,809,366	–	WP_014225043.1, glycosyl transferase	cl01298 Glyco_transf_25 superfamily	–
BFO_RS07410, BFO_1700	1,809,356–1,810,438	–	WP_014225044.1, glycosyl transferase	cl10013 Glycosyltransferase_GTB_type	GT4
BFO_RS14425, BFO_1705	1,812,769–1,814,883	–	WP_052299248.1, hypothetical protein	cd03801 GT1_YqgM_like	GT4
PGL_5					
BFO_RS08625, BFO_1977	2,106,020–2,107,996	–	WP_014225302.1, hypothetical protein	cl28208 RfaB superfamily	–
BFO_RS08630, BFO_1978	2,108,002–2,108,745	–	WP_014225303.1, glycosyl transferase family 2	cd04179 DPM_DPG-synthase_like	GT2
BFO_RS14090, BFO_1987	2,122,302–2,123,087	+	WP_052299260.1, hypothetical protein	cd04186 GT_2_like_c	GT2
BFO_RS08670, BFO_	2,123,084–2,124,346	+	WP_041590821.1, hypothetical protein	cl10013 Glycosyltransferase_GTB_type	GT4
BFO_RS08675, BFO_1990	2,124,694–2,126,031	+	WP_014225312.1, glycosyltransferase group 1 family protein	cl10013 Glycosyltransferase_GTB_type	GT4
BFO_RS08680, BFO_1989	2,126,026–2,127,159	–	WP_014225313.1, glycosyl transferase	cl10013 Glycosyltransferase_GTB_type	GT4
PGL_6					
BFO_RS10550, BFO_2565	2,598,381–2,599,619	–	WP_014225708.1, hypothetical protein	cl10013 Glycosyltransferase_GTB_type	GT4
BFO_RS10555, BFO_2566	2,599,616–2,600,713	–	WP_014225709.1, UDP-N-acetylglucosamine 2-epimerase (non-hydrolyzing)	cd03786 GT1_UDP-GlcNAc_2-Epimerase	–
BFO_RS10600, BFO_2575	2,607,474–2,608,256	+	WP_014225718.1 glycosyl transferase	cd04179 DPM_DPG-synthase_like	GT2

### Codon usage analysis

The presence or absence of certain genes from the genomes of *T. forsythia* and *Tannerella* sp. BU063 may explain pathogenicity of the former and association with periodontal health of the latter. However, it is also possible that different expression levels of orthologous genes found in both genomes were responsible for the disease status. Further, genes that are highly expressed in *T. forsythia* may be assumed to be important for the species. For a number of microorganisms it has been shown that expression levels of individual genes can be predicted based on the differential usage of synonymous codons within the genes [42, 43]. Whereas compositional constraints such as GC content are believed to be responsible for shaping codon usage in many genomes throughout different domains of life, analysis of codon usage is especially interesting in prokaryotes, where the differential usage of synonymous codons of some genomes has been shown to correlate with the availability of the corresponding tRNAs in the cell. The frequent codon/tRNA pairs are thought to enable fast translation of these regions, whereas rare codon/tRNA pairs can slow down translation and improve accuracy. Together, this is often referred to as “translational optimization”. Based on these findings, different approaches have been developed that try to predict expressivity of a gene, based on the codon usage bias found in its sequence.

**Fig. 7:**
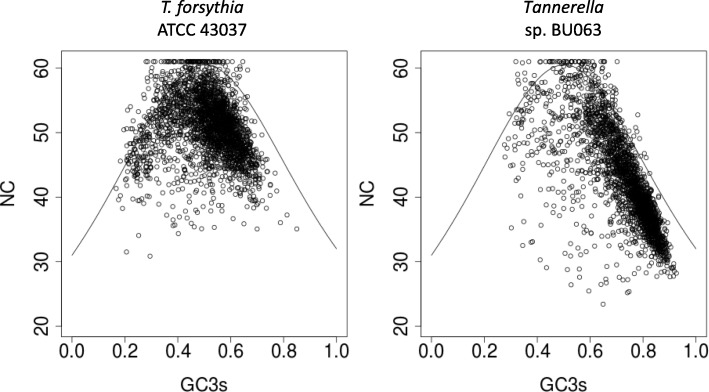
Analysis of codon usage for ATCC 43037 (left panel) and BU063 (right panel). The continuous curves indicate the NC values to be expected for a given GC3s content in the absence of other factors shaping codon usage. Every dot represents a protein coding gene, dots not positioned near the curve therefore represent genes that display a considerable codon usage bias. GC3s: G + C content at synonymous positions, NC: effective number of codons used within the sequence of a gene

We analysed the effective number of codons (Nc) values for the genomes of *T. forsythia* ATCC 43037 and *Tannerella* sp. BU063 and their relationship to the frequency of G and C at synonymous 3rd codon positions (GC3s-content) (Fig. 7). The maximum possible Nc value is 61, as it represents a case whereby all 61 (non-stop) codons are used equally. This is only possible at balanced GC3s content; deviations from that balance result in lower maximum possible Nc values. Our analysis indicated a bias in codon usage for both *T. forsythia* ATCC 43037 and *Tannerella* sp. BU063 (Additional file 5: Tables S5, Additional file 6: Table S6, Additional file 7: Table S7 and Additional file 8: Table S8). While for many genes the Nc value is roughly in the predicted range, numerous genes display a codon usage bias that cannot be explained by compositional constraints alone and, hence, may be explained by translational optimization. In the absence of knowledge on gene expression levels in *T. forsythia*, we used two self-consistent indices, self-consistent Codon Adapation Index (scCAI) [44] and self-consistent normalized Relative Codon Adaption (scnRCA) [45], in combination with criteria that can be applied to the results of both approaches to predict the nature of this bias [46]. scCAI detected the most prominent codon usage bias, predicted to be shaped by GC3s content, in both *T. forsythia* and *Tannerella* sp. BU063 with content criteria values of 0.85 and 0.89, respectively, both above the proposed threshold of 0.7 (Additional file 5: Table S5, Additional file 7: Table S7). The similar scnRCA index aims at predicting a potential translational bias. In case of *T. forsythia,* the scnRCA method was able to remove the influence of the GC3s bias on the analysis as indicated by a content criterion value of 0.56 (Additional file 6: Table S6, Additional file 8: Table S8). However, a content criterion value > 0.5 is suggested to indicate a bias shaped by a GC skew. Two criteria that would indicate a translational bias, the ribosomal criterion and the strength criterion, were both negative. For *Tannerella* sp. BU063*,* scnRCA was not able to remove the GC3s bias sufficiently, as the remaining bias is still predicted to be shaped by GC3s content (content criterion > 0.7). This matches the observation of a higher GC3s content in *Tannerella* sp. BU063 when compared to *T. forsythia* (Fig. 7). We conclude that compositional constraints are the main factors shaping the codon usage bias in both *T. forsythia* and *Tannerella* sp. BU063*.* Whether translational optimization is also a factor shaping the biases in one or both of the genomes remains to be elucidated.

## Discussion

We were able to assemble 99% of the *T. forsythia* ATCC 43037 genome into only three fragments by using the existing contigs generated by Friedrich et al. [20] and new mate-pair data of about 800-fold genome coverage. For obtaining an assembly in one uninterrupted sequence for the *T. forsythia* reference strain the use of sequencing technologies that provide medium-sized or long reads will be required since short-reads, even at very high coverage, were not sufficient to close all gaps. This finding is consistent with the fact that the two completely contiguous *T. forsythia* assemblies for strains KS16 and 3313 had been generated employing Sanger sequencing data in combination with short-read data. However, our current assembly result for ATCC 43037 represents an almost complete genome sequence as a valuable resource for *T. forsythia* studies.

In comparative analyses we provided an assessment of the presence or absence of currently known as well as suggested virulence factors in all presently available *T. forsythia* genome assemblies. We may have missed orthologs in a given strain if genes were located in a region of the genome that was not covered by its assembly. However, our results largely extend previous findings by Endo et al. [22], that showed a small subset of the genes included in our analysis to be conserved in strains 92A2 (mistakenly referred to as ATCC 43037 by the authors), KS16, and 3313, as well as in 16 other *T. forsythia* strains. Similarly, our gene numbers for the core genome were slightly higher than previously estimated by Endo et al., who reported a core genome size of 1733 genes. The deviation, however, is to be expected as only three of the 19 *T. forsythia* strains included in their work had complete genome assemblies whereas 16 were incompletely assembled. Hence, a *bona fide* core gene that was located in a region uncovered in one or more of these incomplete assemblies, would not be included; an effect that can be assumed to be stronger, the higher the number of included incomplete genomes is.

We suggested a number of regions that may be considered as pathogenicity islands. It should be noted that the term “pathogenicity island” usually refers to a genomic region containing genes that were introduced by horizontal gene transfer (HGT) [47]. Our approach does not consider the latter requirement, i.e. we did not assess whether the detected regions show traces of HGT. Further, the genes contained within putative pathogenicity islands presented in our work were inferred to be possibly linked to pathogenicity based on their presence in the *T. forsythia* core genome and their absence in *Tannerella* sp. BU063. How many and which of the reported candidate regions represent true pathogenicity islands, in the sense of the common definition, has yet to be discovered and will require experimental verification. In the context of periodontitis research, however, the finding that such a region is involved in pathogenesis is relevant, regardless of whether or not HGT took place.

In strain 92A2 we detected the already known glycosylation locus and confirmed that parts of it were shared throughout numerous *Bacteroidales* species. We speculate that the glycosylation pathway genes that synthesize the core of the glycan are organized in a different and less conserved way than the already described parts that assemble the outer part of the glycan. It should be noted that our approach relied on the current annotation of *T. forsythia* genes, their predicted functions, and their conserved domains. It is therefore possible that some carbohydrate-active genes were not included in the analysis simply because this functionality has not yet been predicted. Especially as research on prokaryotic glycosylation pathways is still vastly expanding, it will be interesting to see if more conclusive results will be obtainable in the future, as the knowledge on protein architecture and the conserved domain functions stored therein increases.

## Conclusion

The involvement of *Tannerella forsythia* in periodontal disease manifests a major challenge to national health systems. In this work, we provide molecular resources which will facilitate future work on *T. forsythia*. We provide an improved genome assembly of the reference type strain *T. forsythia* ATCC 43037, and we define a soft-core genome and an accessory genome of the species. Comprehensive characterization of the *T. forsythia* genome relative to the non-pathogenic isolate *Tannerella* sp. BU063 allowed us to confirm known virulence factors or suggest their re-evaluation, respectively. Importantly, we highlight genes which so far have not been implicated in the pathogenesis of *T. forsythia*. In summary, our work provides new perspectives for work on *Tannerella* biology, for both basic as well as applied research.

## Methods

### DNA source, extraction and quality control

The type strain of *T*. *forsythia* (ATCC 43037 = FDC 338) was obtained from ATCC (Manassas, VA, USA) and grown under anaerobic conditions in brain–heart infusion broth with supplements as described previously [12]. Bacterial DNA was extracted using the GeneElute Bacterial Genomic DNA Kit (Sigma-Aldrich, Vienna, Austria) following the manufacturer’s protocol. The quality of the genomic DNA was checked on a 0.6% standard agarose gel stained with ethidium bromide, and using a NanoDrop ND-1000 spectrophotometer (ThermoFisher, Waltham, MA, USA). Quantification was performed using a Qubit 3.0. fluorometer together with a dsDNA BR assay kit (ThermoFisher, Waltham, MA, USA).

### Mate-pair library preparation and sequencing

Starting from 1 μg of genomic DNA, a mate-pair library was prepared using a Nextera mate-pair library preparation kit (FC-132-1001, Illumina, San Diego, CA, USA) applying the gel-free version of the library preparation protocol, according to the supplier’s instructions. Briefly, the protocol consists of tagmentation, strand displacement, AMPure purification of the strand displacement reaction, and circularization. After linear DNA digestion, circularized DNA was sheared to a size of 300–1000 bp with a Covaris S220 instrument (Covaris, Woburn, MA, USA) and the following settings: 40 s at 20% duty cycle, intensity 50, temperature 6 °C and 200 cycles per burst. Next, sheared DNA fragments containing the biotinylated junction adapter were purified using streptavidin magnetic beads followed by end-repair, A-tailing, and ligation of Illumina adapters to the ends of the DNA fragments. The library was amplified by polymerase chain reaction (PCR) applying the following cycling conditions: initial denaturation at 98 °C/30 s, followed by 10 cycles at 98 °C/10 s, 60 °C/30 s, 72 °C/30 s, and a final extension at 72 °C/5 min. After PCR clean-up, 1 μl of the library was taken for validation using a 2100 Bioanalyzer (Agilent, Santa Clara, CA, USA). Library quantification was accomplished on a Qubit 3.0 fluorometer using a dsDNA BR assay kit, thereafter, the library was sequenced at the VBCF Next Generarion Sequencing core facility (Vienna, Austria) on an Illumina HiSeq 2500 sequencing instrument using v4 sequencing chemistry and a 2 × 125 nt paired-end sequencing protocol.

#### Quality control and filtering of Illumina sequencing data

FastQC v0.11.4 (http://www.bioinformatics.babraham.ac.uk/projects/fastqc/) was used for initial quality checking of raw sequencing reads and to assess the outcome of read filtering procedures. Raw reads from the paired-end library were de-duplicated, considering two read pairs as duplicates if bases 15 to 50 of both the forward and of the reverse reads were identical. The non-redundant reads were then trimmed and filtered with Trimmomatic 0.35 [48], applying the following parameters: LEADING:3 TRAILING:3 SLIDINGWINDOW:4:15 MINLEN:36. Due to remaining potential quality issues the reads were additionally cropped on both ends (15 bases at the head, eight bases at the tail), using the fastx toolkit (http://hannonlab.cshl.edu/fastx_toolkit/). Raw reads from the mate-pair library were cropped to various lengths for scaffolding test runs (only using nucleotides 1–50, 1–80 or 8–106, of each read, respectively); for the final scaffolding procedure the 50 nt long cropped reads were used.

### Assembly scaffolding

SOAPdenovo 2.04 [49] was used for scaffolding. The helper program finalFusion, also maintained by SOAPdenovo developers, was used to prepare the input contigs for applying the scaffolding steps (map-scaff) of the main program. The k-mer size parameter was varied in repetitions of this procedure (K = 33, 43, 45, 47, 49), where K = 47 resulted in an assembly with the highest values for N50 scaffold length, and for the size of the largest scaffold, respectively. Gap-filling, as carried out by SOAPdenovo, was enabled by using the -F parameter. Results were assessed using QUAST v3.2 [50], additionally, critical links were validated by manual inspection of the mate-pairs supporting these links in IGV 2.3.68 [51, 52] and by analysing the amount and mapping positions of mate-pairs supporting these links.

### Phylogenetic analysis

Mash v2.0 [30] was used for distance calculation (programm call “mash dist” with default parameters). Trees were calculated using the Fitch-Margoliash algorithm as implented in PHYLIP v3.6 [53], with global rearrangement and randomizing the input order (10x jumbling). Trees were displayed using Newick utilities [54].

### Whole-genome alignments

Whole-genome alignments of more than two genomes were generated and visualized with Mauve (version snapshot 2015-02-13 build 0) [55], using the progressiveMauve algorithm with default parameters --seed-weight = 15 --gap-open = 400 --gap-extend = 30 --scoring-scheme = sp. The contigs of fragmented assemblies were ordered and oriented using the contiguous genome assembly of *T. forsythia* 92A2 as reference with Mauve’s “reorder contigs” module prior to alignment. Additional alignments employing blastn [56, 57] were used for calculating the fraction of alignable regions to strain 92A2 per genome.

Whole-genome alignments between ATCC 43037 and BU063 were carried out using the MUMmer 3.23 software package [58], employing nucmer with default parameters --mumreference -b 200 --nobanded -c 65 --delta -D 5 -d 0.12 --extend -g 90 -l 20 --optimize --simplify for aligning and mummerplot with the option --filter for creating Gnuplot scripts. Six-frame amino acid translation alignments between ATCC 43037 and BU063 were generated with promer with the parameters -mumreference -b 60 -c 20 -g 30 -l 6 -m 8 -× 2. Gunplot scripts were adapted manually with respect to aesthetics and readability and plotted with Gnuplot 4.4 (http://www.gnuplot.info/).

### Analysis of core- and accessory genomes

The *T. forsythia* core and accessory genomes were compiled using components of the GET_HOMOLOGUES pipeline [59]. All genome assemblies included in the analysis were downloaded as RefSeq gbff files from the NCBI ftp server (ftp://ftp.ncbi.nlm.nih.gov/genomes/) as input for get_homologues.pl. As starting point for further analysis, an all-vs-all blastp [56, 57] of all coding sequences (CDS) contained in the input files was performed in a batchwise manner, using the parameters -dbsize 100,000,000 -seg yes -soft_masking true -evalue 0.01 -outfmt 6 qseqid sseqid pident length qlen slen qstart qend sstart send evalue bitscore -max_target_seqs N (where N is the total number of sequences in the database used in that run). Based on the blastp results, clusters of putative orthologs were clustered with get_homologues.pl in two parallel runs, one employing the OrthoMCL algorithm [60], the other employing the cluster of orthologous groups of proteins (COG) triangles algorithm [61]. Both algorithms infer orthology based on bidirectional best hits (sometimes also referred to as symmetrical best hits or reciprocal best hits). Additional thresholds for two genes to be allowed to group in the same cluster were: sequence identity of at least 30% (−S 30), sequence coverage of the alignment of at least 75% (−C 75), and an Expect (E) value of < 10^− 5^ (−E 1e-05). Clusters were allowed to contain genes from any number of the included genomes (−t 0). For further parameters the default values -c 0 -z 0 -I 0 -m local -n 2 -M 0 -G 1 -P 0 -F 1.5 -N 0 -B 50 -b 0 -s 0 -D 0 -g 0 -a ‘0’ -× 0 -R 0 -A 0 were used. The genome of strain 92A2 was used as reference (−r), which, however, only has an influence on the names given to the resulting clusters when using OrthoMCL or COG triangles as clustering method. The intersection of the cluster sets generated by the two different algorithms was extracted using compare_clusters.pl and used for further analysis. Orthology clusters containing genes from all *T. forsythia* genomes were extracted using the script parse_pangenome_matrix.pl; the genes contained in these clusters constitute the core genome of *T. forsythia.* In an additional run, orthologs were required to be present in at least 80% of the *T. forsythia* genomes to become part of a relaxed form of the core genome, sometimes referred to as “soft core genome”. Allowing an ortholog to be absent in one of the assemblies reduces the risk of incorrectly excluding bona fide core genes from the core genome due to annotation, assembly or sequencing errors, or the incomplete nature of some of the included assemblies. *Tannerella forsythia* core and pan genome sizes were estimated based on random sampling by using only the *T. forsythia* genomes as input for get_homologues.pl with the additional parameter -c. Plots illustrating these estimations were generated with plot_pancore_matrix.pl, using the parameter -f core_Tettelin for the core genome plot and -f pan for the pangenome plot. Comparisons on the presence or absence of single genes were carried out using the script check_BDBHs.pl. All these Perl scripts are part of the GET_HOMOLOGUES pipeline.

### Detection of putative pathogenicity islands

Based on the results of the pan-genome analysis, putative pathogenicity islands were detected as follows: Genes that were found to be present in at least eight of the ten *T. forsythia* strains but absent from *Tannerella* sp. BU063 were considered to be *T. forsythia*-specific and assessed for their co-localization within the genome of strain 92A2 using the Perl script GeneClusterFinder.pl developed in this work. The script takes a file containing all annotated genes of the genome in tabular form (as can be downloaded from https://www.ncbi.nlm.nih.gov/genome/proteins/11045?genome_assembly_id=231734) as reference input file, assigning sequential numbers to the genes sorted by position. This step is necessary, as the locus tags used by NCBI are not necessarily sequential. A file containing the locus tags of all *T. forsythia*-specific genes is then loaded as second input and the corresponding sequential numbers are extracted. Finally, stretches of consecutive numbers are searched in the resulting set of numbers.

### Searching for glycosylation loci

Putative glycosylation loci were defined as genomic regions where at least three predicted Gtfs or other putative glycosylation-related genes occur within a stretch of 15 consecutive genes. The current Reference Sequence (RefSeq) gene set for strain 92A2 was used as reference. The complete gene set was annotated using the carbohydrate-active enzyme specific annotation web server dbCAN [62]. Additionally, conserved domains were annotated for all genes, using NCBI’s CD-Search [63–66] (parameters: database: CDD – 53,069 PSSMs, expect value threshold 0.01, composition-corrected scoring on, low-complexity filter off, maximum number of hits 500, include retired sequences on). All genes that either were predicted as Gtfs by dbCAN or predicted to contain a Gtf-associated conserved domain by CD-Search were combined and used for the subsequent steps. Analysing the co-localization of these genes employed a custom Perl script implementing a sliding-window approach to find stretches of 15 consecutive genes encompassing at least three putative Gtfs. Redundant windows were removed and overlapping ones combined; the split region of the circular genome in the assembly was checked manually. Additional analysis on the presence or absence of the detected putative glycosylation loci in other members of the *Bacteroidales* order was performed using Gecko 3.1 [67]. Organisms included in this analysis were chosen based on previous work [11], the RefSeq assembly versions of the corresponding genomes were downloaded from the NCBI ftp server as GenBank flat files (Additional file 4: Table S4) and used as input for the script gecko3_gb_to_transclust_to_cog.py from the Gecko suite. First, this script was used to prepare a Blastp search (version 2.2.30+) by using the parameter -prepareSingleBlast. This sets up a database and a query FASTA file containing all CDS from all included genomes and executes the corresponding Blastp command with an E-value cut-off of 0.01. The file containing the Blastp results as well as the query file described above were loaded into Transclust 1.0 [68]. Clustering was performed using Best Hit (BeH) as cost model and a density parameter of 40. The results produced by Transclust were converted into the format required as input for Gecko, using the script gecko3_gb_to_transclust_to_cog.py with the parameter -transclustToCog. Whether a putative glycosylation locus or parts of it can be found in one of the included genomes apart from *T. forsythia* was checked as follows: The homology IDs assigned to the genes constituting such a locus during data preparation were extracted and used as query cluster in a “manual cluster” search in Gecko 3.1. The number of genes required in a cluster to be detected was initially set to the number of genes in the query minus one and the maximum distance between genes was set to 15. If this did not detect the cluster or parts of it in non-*T. forsythia* genomes, additional searches were carried out with more relaxed parameters, i.e. setting the maximum distance between genes to 30 and reducing the minimum number of genes required in a cluster to be detected. The value for the latter parameter was decreased by one in each subsequent run either until parts of the cluster were found in a non-*T. forsythia* strain or until the value was two.

### Codon usage analysis

Nc values [69] and GC3s values were calculated with CodonW using the parameters -all_indices -nomenu (http://codonw.sourceforge.net//culong.html). Fasta files containing the nucleotide sequences of all CDS of the respective genome were downloaded from NCBI’s ftp server and all CDS for which the “pseudo” qualifier was set to “true” were eliminated prior to the analysis. scCAI values and scnRCA values were calculated using the program scnRCA with the parameters -g true -d 2.0 -p 1.0 -m − 1 and GenBank flat files as input files [44, 45].

## Supplementary information

**Additional file 1: Table S1.** xBlast Score Ratio values of 45 suggested virulence genes blasted against entire *Tannerella* genomes (heatmap plot in Fig. 4).

**Additional file 2: Table S2.** Blast Score Ratio values of 45 suggested virulence genes blasted against annotated *Tannerella* CDS (heatmap plot in Additional file 11: Figure S2).

**Additional file 3: Table S3.** List of promising therapeutic targets based on presence-absence analyses.

**Additional file 4: Table S4.** *Bacteroidales* genome assemblies used for the identification of putative glycosylation loci.

**Additional file 5: Table S5.** Codon usage bias (scCAI). Top 20 genes of ATCC 43037 (a) and *Tannerella sp.* BU063 (b) showing the highest scCAI values. Only functionally annotated proteins were selected.

**Additional file 6: Table S6.** Codon usage bias (scnRCA). Top 20 genes of ATCC 43037 (a) and *Tannerella sp.* BU063 (b) showing the highest scnRCA values. Only functionally annotated proteins were selected.

**Additional file 7: Table S7.** Codon usage bias (scCAI) including “hypothetical proteins”. Top 20 genes of ATCC 43037 (a) and *Tannerella sp.* BU063 (b) showing the highest scCAI values.

**Additional file 8: Table S8.** Codon usage bias (scnRCA) including “hypothetical proteins”. Top 20 genes of ATCC 43037 (a) and *Tannerella sp.* BU063 (b) showing the highest scnRCA values.

**Additional file 9: Table S9.** Pathogenicity islands in *T. forsythia* strain 92A2 as inferred from comparisons to *Tannerella* sp. BU063 and positions of genes encoded therein including their functional annotation.

**Additional file 10: Figure S1.** Span size distribution of the mate-pair library prepared from DNA of *T. forsythia* strain ATCC 43037. The peak of the distribution is at 1759 bp, indicated by the red line.

**Additional file 11: Figure S2.** Blast Score Ratio values plotted as heatmap for 45 suggested virulence genes in ten *T. forsythia* strains and the genome of a putative health-associated *Tannerella* sp. BU063. In contrast to Fig. 4, the 45 gene sequences were blasted against sequences of annotated CDS in each genome.

**Additional file 12: File S1.** Alignments of the sequence KP715369 to *T. forsythia* genomes.

**Additional file 13: File S2.** Detailed view on the alignment of *T. forsythia* karilysin to a putative orthologue in *Tannerella* sp. BU063.

## Data Availability

The improved genome assembly for *T. forsythia* ATCC 43037 has been deposited at DDBJ/ENA/GenBank under the accession VFJI00000000. The version described in this paper is version VFJI01000000. Mate-pairs of *T. forsythia* ATCC 43037 were deposited in the Sequence Read Archive under accession SRR9302598 (BioProject PRJNA548889, BioSample SAMN12058270).

## Abbreviations

ATCC: American Type Culture Collection
BeH: Best hit
BSR: BLAST score ratio
CDS: Coding sequence
COG: Cluster of orthologous groups of proteins
CTD: C-terminal domain
E-value: Expect value
GC3s: G and C at synonymous 3rd codon positions
Gtf: Glycosyltransferase
HGT: Horizontal gene transfer
IgSF: Immunoglobulin-superfamily
kbp: Kilobasepair
KEGG: Kyoto encyclopedia of genes and genomes
Mbp: Megabasepair
Nc: Effective number of codons
NCBI: National Center for Biotechnology Information
nt: Nucleotides
PCR: Polymerase chain reaction
PUS: Polysaccharide utilization locus
RefSeq: Reference Sequence
scCAI: Self-consistent Codon Adapation Index
scnRCA: Self-consistent normalized Relative Codon Adaption
S-layer: Surface layer
T9SS: Type IX secretion system
