# Supplementary material for: Comparative genome characterization of the periodontal pathogen Tannerella forsythia
Source: BMC Genomics. 2020 Feb 11;21:150. doi: 10.1186/s12864-020-6535-y (PMC7014623; doi:10.1186/s12864-020-6535-y)
Supplement: Supplementary file 6 — Additional file 6: Table S6. Codon usage bias (scnRCA). Top 20 genes of ATCC 43037 (a) and Tannerella sp. BU063 (b) showing the highest scnRCA values. Only functionally annotated proteins were selected. [file 12864_2020_6535_MOESM6_ESM.doc]

(a)

| **Locus tag** | **Protein ID** | **GC3s** | **scnRCA** | **Annotated function** |
| --- | --- | --- | --- | --- |
| Tanf_RS13395 | WP_014225575.1 | 0.35 | 0.661 | rubredoxin |
| Tanf_RS11020 | WP_014223794.1 | 0.49 | 0.650 | transcriptional regulator |
| Tanf_RS09755 | WP_014224806.1 | 0.57 | 0.643 | membrane protein |
| Tanf_RS04190 | WP_014225182.1 | 0.65 | 0.641 | RidA family protein |
| Tanf_RS03805 | WP_014225089.1 | 0.54 | 0.641 | thiol reductase thioredoxin |
| Tanf_RS10420 | WP_046825825.1 | 0.51 | 0.640 | 50S ribosomal protein L25/general stress protein Ctc |
| Tanf_RS03320 | WP_046824914.1 | 0.60 | 0.637 | succinate dehydrogenase |
| Tanf_RS07515 | WP_046825440.1 | 0.53 | 0.634 | 50S ribosomal protein L25/general stress protein Ctc |
| Tanf_RS08940 | WP_046825668.1 | 0.69 | 0.633 | diphosphate--fructose-6-phosphate 1-phosphotransferase |
| Tanf_RS11615 | WP_046825980.1 | 0.64 | 0.632 | saccharopine dehydrogenase |
| Tanf_RS12655 | WP_014226038.1 | 0.54 | 0.630 | arginine decarboxylase |
| Tanf_RS13385 | WP_046826198.1 | 0.49 | 0.629 | DNA starvation/stationary phase protection protein |
| Tanf_RS01585 | WP_046824662.1 | 0.65 | 0.627 | phosphonate ABC transporter ATP-binding protein |
| Tanf_RS12345 | WP_046826068.1 | 0.68 | 0.627 | phosphoenolpyruvate carboxykinase (ATP) |
| Tanf_RS00225 | WP_041591316.1 | 0.47 | 0.625 | peptidylprolyl isomerase |
| Tanf_RS08125 | WP_014226426.1 | 0.44 | 0.624 | 50S ribosomal protein L9 |
| Tanf_RS00500 | WP_046824514.1 | 0.62 | 0.623 | β-ketoacyl-ACP reductase |
| Tanf_RS00640 | WP_046824529.1 | 0.61 | 0.622 | NADH:ubiquinone reductase (Na(+)-transporting) subunit F |
| Tanf_RS08955 | WP_014223696.1 | 0.58 | 0.619 | phosphoglycerate kinase |
| Tanf_RS00630 | WP_014225454.1 | 0.56 | 0.618 | NADH:ubiquinone reductase (Na(+)-transporting) subunit D |

(b)

| **Locus tag** | **Protein ID** | **GC3s** | **scnRCA** | **Annotated function** |
| --- | --- | --- | --- | --- |
| BCB71_RS09850 | WP_037996200.1 | 0.76 | 0.690 | phosphoserine transaminase |
| BCB71_RS09845 | WP_037981487.1 | 0.72 | 0.687 | 3-phosphoglycerate dehydrogenase |
| BCB71_RS00425 | WP_069174653.1 | 0.76 | 0.677 | 50S ribosomal protein L25/general stress protein Ctc |
| BCB71_RS01780 | WP_037985925.1 | 0.68 | 0.667 | DNA starvation/stationary phase protection protein |
| BCB71_RS04615 | WP_069176361.1 | 0.85 | 0.667 | pseudouridine synthase |
| BCB71_RS00035 | WP_069174598.1 | 0.82 | 0.666 | methylaspartate ammonia-lyase |
| BCB71_RS11610 | WP_037982229.1 | 0.87 | 0.665 | DNA-binding response regulator |
| BCB71_RS00005 | WP_037980691.1 | 0.75 | 0.661 | 50S ribosomal protein L31 |
| BCB71_RS09985 | WP_069175987.1 | 0.79 | 0.661 | 30S ribosomal protein S1 |
| BCB71_RS08955 | WP_069175852.1 | 0.74 | 0.661 | 30S ribosomal protein S18 |
| BCB71_RS09105 | WP_038012240.1 | 0.76 | 0.660 | molecular chaperone GroEL |
| BCB71_RS10790 | WP_038001575.1 | 0.78 | 0.656 | methylmalonyl-CoA carboxyltransferase |
| BCB71_RS03600 | WP_069175075.1 | 0.75 | 0.653 | peroxiredoxin |
| BCB71_RS06720 | WP_069175536.1 | 0.78 | 0.651 | formate C-acetyltransferase |
| BCB71_RS10520 | WP_069176067.1 | 0.86 | 0.649 | glycine cleavage system protein T |
| BCB71_RS08950 | WP_037982859.1 | 0.72 | 0.649 | 50S ribosomal protein L9 |
| BCB71_RS06255 | WP_069175474.1 | 0.82 | 0.647 | pyruvate:ferredoxin (flavodoxin) oxidoreductase |
| BCB71_RS01110 | WP_069174738.1 | 0.83 | 0.646 | 1-pyrroline-5-carboxylate dehydrogenase |
| BCB71_RS04470 | WP_069175203.1 | 0.78 | 0.641 | molecular chaperone DnaK |
| BCB71_RS05095 | WP_069175296.1 | 0.80 | 0.641 | glutamine--tRNA ligase |
