# Supplementary material for: Comparative genome characterization of the periodontal pathogen Tannerella forsythia
Source: BMC Genomics. 2020 Feb 11;21:150. doi: 10.1186/s12864-020-6535-y (PMC7014623; doi:10.1186/s12864-020-6535-y)
Supplement: Supplementary file 7 — Additional file 7: Table S7. Codon usage bias (scCAI) including “hypothetical proteins”. Top 20 genes of ATCC 43037 (a) and Tannerella sp. BU063 (b) showing the highest scCAI values. [file 12864_2020_6535_MOESM7_ESM.doc]

(a)

| **Locus tag** | **Protein ID** | **GC3s** | **scCAI** | **Annotated function** |
| --- | --- | --- | --- | --- |
| Tanf_RS04800 | WP_046825063.1 | 0.79 | 0.686 | hypothetical protein |
| Tanf_RS08570 | WP_046825564.1 | 0.70 | 0.674 | glutamate formiminotransferase |
| Tanf_RS13445 | WP_052449128.1 | 0.74 | 0.668 | DUF5115 domain-containing protein |
| Tanf_RS08940 | WP_046825668.1 | 0.69 | 0.663 | diphosphate--fructose-6-phosphate 1-phosphotransferase |
| Tanf_RS04270 | WP_046825006.1 | 0.54 | 0.654 | hypothetical protein |
| Tanf_RS12675 | WP_046826108.1 | 0.69 | 0.650 | glycine cleavage system protein T |
| Tanf_RS08590 | WP_014224209.1 | 0.75 | 0.647 | histidine ammonia-lyase |
| Tanf_RS12345 | WP_046826068.1 | 0.68 | 0.645 | phosphoenolpyruvate carboxykinase (ATP) |
| Tanf_RS13435 | WP_014225566.1 | 0.69 | 0.643 | RagB/SusD family nutrient uptake outer membrane protein |
| Tanf_RS13820 | WP_052449037.1 | 0.85 | 0.640 | DUF1566 domain-containing protein |
| Tanf_RS09840 | WP_014224788.1 | 0.71 | 0.639 | Fe-S assembly SUF system protein |
| Tanf_RS03690 | WP_041591207.1 | 0.69 | 0.639 | methylglyoxal synthase |
| Tanf_RS02790 | WP_046824839.1 | 0.66 | 0.635 | hypothetical protein |
| Tanf_RS08945 | WP_014223698.1 | 0.73 | 0.632 | riboflavin synthase |
| Tanf_RS12300 | WP_046826083.1 | 0.68 | 0.630 | methionine--tRNA ligase |
| Tanf_RS10925 | WP_046825875.1 | 0.71 | 0.626 | α-mannosidase |
| Tanf_RS11615 | WP_046825980.1 | 0.64 | 0.626 | saccharopine dehydrogenase |
| Tanf_RS01585 | WP_046824662.1 | 0.65 | 0.625 | phosphonate ABC transporter ATP-binding protein |
| Tanf_RS13430 | WP_046826204.1 | 0.69 | 0.620 | SusC/RagA family TonB-linked outer membrane protein |
| Tanf_RS08550 | WP_046825561.1 | 0.69 | 0.615 | urocanate hydratase |

(b)

| **Locus tag** | **Protein ID** | **GC3s** | **scCAI** | **Annotated function** |
| --- | --- | --- | --- | --- |
| BCB71_RS03555 | WP_069176350.1 | 0.92 | 0.710 | dTDP-4-dehydrorhamnose reductase |
| BCB71_RS09195 | WP_069175880.1 | 0.93 | 0.687 | ribulose-phosphate 3-epimerase |
| BCB71_RS00395 | WP_069174648.1 | 0.90 | 0.680 | AAA family ATPase |
| BCB71_RS02395 | WP_037985240.1 | 0.91 | 0.675 | DNA topoisomerase IV subunit B |
| BCB71_RS10595 | WP_069176081.1 | 0.91 | 0.661 | nucleoside hydrolase |
| BCB71_RS06855 | WP_069175560.1 | 0.92 | 0.657 | GDP-fucose synthetase |
| BCB71_RS02720 | WP_069174956.1 | 0.92 | 0.655 | acyl-CoA dehydrogenase |
| BCB71_RS04230 | WP_069175161.1 | 0.91 | 0.655 | β-ketoacyl synthase |
| BCB71_RS08115 | WP_069175739.1 | 0.85 | 0.653 | UDP-N-acetylenolpyruvoylglucosamine reductase |
| BCB71_RS00625 | WP_069174678.1 | 0.87 | 0.650 | tRNA (adenosine(37)-N6)-threonylcarbamoyltransferase complex ATPase subunit type 1 TsaE |
| BCB71_RS03595 | WP_069175074.1 | 0.89 | 0.648 | cystathionine β-lyase |
| BCB71_RS09465 | WP_069175912.1 | 0.89 | 0.648 | coproporphyrinogen III oxidase |
| BCB71_RS10015 | WP_069175992.1 | 0.92 | 0.647 | prephenate dehydratase |
| BCB71_RS10385 | WP_069176049.1 | 0.89 | 0.644 | 3-deoxy-manno-octulosonate cytidylyltransferase |
| BCB71_RS11170 | WP_037986571.1 | 0.86 | 0.639 | 5-nitroimidazole antibiotic resistance protein |
| BCB71_RS09640 | WP_069175937.1 | 0.90 | 0.637 | glycosyl transferase family 2 |
| BCB71_RS03565 | WP_069175069.1 | 0.89 | 0.635 | nicotinate phosphoribosyltransferase |
| BCB71_RS11610 | WP_037982229.1 | 0.87 | 0.633 | DNA-binding response regulator |
| BCB71_RS05630 | WP_037984697.1 | 0.88 | 0.630 | hypothetical protein |
| BCB71_RS03550 | WP_069175067.1 | 0.87 | 0.629 | peptide chain release factor 3 |
