# Supplementary material for: Comparative genome characterization of the periodontal pathogen Tannerella forsythia
Source: BMC Genomics. 2020 Feb 11;21:150. doi: 10.1186/s12864-020-6535-y (PMC7014623; doi:10.1186/s12864-020-6535-y)
Supplement: Supplementary file 8 — Additional file 8: Table S8. Codon usage bias (scnRCA) including “hypothetical proteins”. Top 20 genes of ATCC 43037 (a) and Tannerella sp. BU063 (b) showing the highest scnRCA values. [file 12864_2020_6535_MOESM8_ESM.doc]

(a)

| **Locus tag** | **Protein ID** | **GC3s** | **scnRCA** | **Annotated function** |
| --- | --- | --- | --- | --- |
| Tanf_RS13395 | WP_014225575.1 | 0.35 | 0.661 | rubredoxin |
| Tanf_RS11020 | WP_014223794.1 | 0.49 | 0.650 | transcriptional regulator |
| Tanf_RS04270 | WP_046825006.1 | 0.54 | 0.644 | hypothetical protein |
| Tanf_RS09755 | WP_014224806.1 | 0.57 | 0.643 | membrane protein |
| Tanf_RS04190 | WP_014225182.1 | 0.65 | 0.641 | RidA family protein |
| Tanf_RS03805 | WP_014225089.1 | 0.54 | 0.641 | thiol reductase thioredoxin |
| Tanf_RS10420 | WP_046825825.1 | 0.51 | 0.640 | 50S ribosomal protein L25/general stress protein Ctc |
| Tanf_RS03320 | WP_046824914.1 | 0.60 | 0.637 | succinate dehydrogenase |
| Tanf_RS07515 | WP_046825440.1 | 0.53 | 0.634 | 50S ribosomal protein L25/general stress protein Ctc |
| Tanf_RS08940 | WP_046825668.1 | 0.69 | 0.633 | diphosphate--fructose-6-phosphate 1-phosphotransferase |
| Tanf_RS11615 | WP_046825980.1 | 0.64 | 0.632 | saccharopine dehydrogenase |
| Tanf_RS08055 | WP_046825502.1 | 0.52 | 0.631 | hypothetical protein |
| Tanf_RS12655 | WP_014226038.1 | 0.54 | 0.630 | arginine decarboxylase |
| Tanf_RS13385 | WP_046826198.1 | 0.49 | 0.629 | DNA starvation/stationary phase protection protein |
| Tanf_RS01585 | WP_046824662.1 | 0.65 | 0.627 | phosphonate ABC transporter ATP-binding protein |
| Tanf_RS12345 | WP_046826068.1 | 0.68 | 0.627 | phosphoenolpyruvate carboxykinase (ATP) |
| Tanf_RS00225 | WP_041591316.1 | 0.47 | 0.625 | peptidylprolyl isomerase |
| Tanf_RS08125 | WP_014226426.1 | 0.44 | 0.624 | 50S ribosomal protein L9 |
| Tanf_RS00500 | WP_046824514.1 | 0.62 | 0.623 | β-ketoacyl-ACP reductase |
| Tanf_RS00640 | WP_046824529.1 | 0.61 | 0.622 | NADH:ubiquinone reductase (Na(+)-transporting) subunit F |

(b)

| **Locus tag** | **Protein ID** | **GC3s** | **scnRCA** | **Annotated function** |
| --- | --- | --- | --- | --- |
| BCB71_RS09850 | WP_037996200.1 | 0.763 | 0.6899583802300218 | phosphoserine transaminase |
| BCB71_RS09845 | WP_037981487.1 | 0.722 | 0.6871002675655838 | 3-phosphoglycerate dehydrogenase |
| BCB71_RS10175 | WP_038010704.1 | 0.767 | 0.6793331493405266 | hypothetical protein |
| BCB71_RS00425 | WP_069174653.1 | 0.758 | 0.6771871871198151 | 50S ribosomal protein L25/general stress protein Ctc |
| BCB71_RS01780 | WP_037985925.1 | 0.679 | 0.6670426250980184 | DNA starvation/stationary phase protection protein |
| BCB71_RS04615 | WP_069176361.1 | 0.852 | 0.6668751668092145 | pseudouridine synthase |
| BCB71_RS00035 | WP_069174598.1 | 0.823 | 0.666352827031594 | methylaspartate ammonia-lyase |
| BCB71_RS11610 | WP_037982229.1 | 0.866 | 0.6652384173830138 | DNA-binding response regulator |
| BCB71_RS00005 | WP_037980691.1 | 0.753 | 0.6613433458324903 | 50S ribosomal protein L31 |
| BCB71_RS09985 | WP_069175987.1 | 0.790 | 0.6611464055550221 | 30S ribosomal protein S1 |
| BCB71_RS08955 | WP_069175852.1 | 0.742 | 0.6606482919857061 | 30S ribosomal protein S18 |
| BCB71_RS09105 | WP_038012240.1 | 0.755 | 0.660345180459216 | molecular chaperone GroEL |
| BCB71_RS10790 | WP_038001575.1 | 0.776 | 0.6562648456289142 | methylmalonyl-CoA carboxyltransferase |
| BCB71_RS03600 | WP_069175075.1 | 0.751 | 0.6534344988898249 | peroxiredoxin |
| BCB71_RS02765 | WP_037985282.1 | 0.710 | 0.6521341559309617 | hypothetical protein |
| BCB71_RS06720 | WP_069175536.1 | 0.780 | 0.6509665675077729 | formate C-acetyltransferase |
| BCB71_RS10520 | WP_069176067.1 | 0.860 | 0.6491504076993898 | glycine cleavage system protein T |
| BCB71_RS08950 | WP_037982859.1 | 0.721 | 0.6487716280218463 | 50S ribosomal protein L9 |
| BCB71_RS06255 | WP_069175474.1 | 0.821 | 0.647131046950351 | pyruvate:ferredoxin (flavodoxin) oxidoreductase |
| BCB71_RS01110 | WP_069174738.1 | 0.827 | 0.6458411415739095 | 1-pyrroline-5-carboxylate dehydrogenase |
