# Supplementary material for: Comparative genome characterization of the periodontal pathogen Tannerella forsythia
Source: BMC Genomics. 2020 Feb 11;21:150. doi: 10.1186/s12864-020-6535-y (PMC7014623; doi:10.1186/s12864-020-6535-y)
Supplement: Supplementary file 9 — Additional file 9: Table S9. Pathogenicity islands in T. forsythia strain 92A2 as inferred from comparisons to Tannerella sp. BU063 and positions of genes encoded therein including their functional annotation. [file 12864_2020_6535_MOESM9_ESM.doc]

| **Start** | **Stop** | **Strand** | **Locus tag** | **Protein name** |
| --- | --- | --- | --- | --- |
| **PP_island_1** | | | | |
| 34748 | 37270 | - | BFO_RS00145 | polysaccharide deacetylase |
| 37320 | 39122 | - | BFO_RS00150 | SusD/RagB family nutrient-binding outer membrane lipoprotein |
| 39153 | 42347 | - | BFO_RS00155 | SusC/RagA family TonB-linked outer membrane protein |
| 42420 | 43274 | - | BFO_RS00160 | hypothetical protein |
| 43302 | 45182 | - | BFO_RS00165 | SusD/RagB family nutrient-binding outer membrane lipoprotein |
| 45202 | 48300 | - | BFO_RS00170 | SusC/RagA family TonB-linked outer membrane protein |
| 48637 | 49491 | - | BFO_RS00175 | hypothetical protein |
| **PP_island _2** | | | | |
| 226054 | 226602 | + | BFO_RS00905 | DNA-directed RNA polymerase sigma-70 factor |
| 226602 | 226883 | + | BFO_RS00910 | hypothetical protein |
| 226979 | 227257 | + | BFO_RS00915 | hypothetical protein |
| 227271 | 228506 | + | BFO_RS00920 | hypothetical protein |
| 228935 | 230497 | + | BFO_RS00925 | hypothetical protein |
| 230500 | 231063 | + | BFO_RS00930 | hypothetical protein |
| 231060 | 232550 | + | BFO_RS00935 | hypothetical protein |
| 232547 | 233980 | + | BFO_RS00940 | CRISPR-associated RAMP protein |
| 233980 | 234357 | + | BFO_RS00945 | TIGR04423 family type III CRISPR-associated protein |
| **PP_island_3** | | | | |
| 301209 | 301682 | + | BFO_RS01250 | histidinol phosphate phosphatase |
| 301825 | 302223 | + | BFO_RS01255 | MULTISPECIES: hypothetical protein |
| 302265 | 302825 | + | BFO_RS01260 | hypothetical protein |
| 302884 | 303897 | + | BFO_RS14320 | DUF805 domain-containing protein |
| 303906 | 305135 | + | BFO_RS01270 | hypothetical protein |
| 305166 | 305849 | + | BFO_RS01275 | PorT family protein |
| 305921 | 306187 | + | BFO_RS01280 | hypothetical protein |
| 306210 | 306536 | + | BFO_RS01285 | RteC protein |
| **PP_island_4** | | | | |
| 703118 | 706315 | + | BFO_RS02900 | hypothetical protein |
| 706340 | 707200 | - | BFO_RS02905 | ATP synthase F1 subunit gamma |
| 707229 | 708821 | - | BFO_RS02910 | F0F1 ATP synthase subunit alpha |
| 708854 | 709432 | - | BFO_RS02915 | ATP synthase F1 subunit delta |
| 709500 | 709994 | - | BFO_RS02920 | ATP synthase F0 subunit B |
| 710012 | 710263 | - | BFO_RS02925 | ATP synthase F0 subunit C |
| 710304 | 711353 | - | BFO_RS02930 | ATP synthase F0 subunit A |
| 711376 | 711816 | - | BFO_RS02935 | hypothetical protein |
| 711845 | 712090 | - | BFO_RS02940 | hypothetical protein |
| 712095 | 713618 | - | BFO_RS02945 | F0F1 ATP synthase subunit beta |
| 713871 | 715178 | - | BFO_RS02950 | peptidase S41 |
| **PP_island_5** | | | | |
| 880420 | 880674 | + | BFO_RS03570 | hypothetical protein |
| 880741 | 881451 | - | BFO_RS03575 | hypothetical protein |
| 881455 | 886140 | - | BFO_RS03580 | ABC transporter ATP-binding protein |
| 886137 | 889223 | - | BFO_RS03585 | AcrB/AcrD/AcrF family protein |
| 889275 | 890342 | - | BFO_RS03590 | efflux RND transporter periplasmic adaptor subunit |
| 890373 | 891884 | - | BFO_RS03595 | TolC family protein |
| 891927 | 895286 | - | BFO_RS03600 | xanthan lyase |
| 895311 | 896207 | - | BFO_RS03605 | ABC transporter ATP-binding protein |
| **PP_island_6** | | | | |
| 969513 | 970010 | - | BFO_RS03885 | hypothetical protein |
| 970125 | 970622 | - | BFO_RS03890 | hypothetical protein |
| 970632 | 971684 | - | BFO_RS03895 | hypothetical protein |
| 972036 | 974807 | + | BFO_RS03900 | S9 family peptidase |
| 974892 | 975470 | + | BFO_RS03905 | RNA polymerase sigma-70 factor |
| 975544 | 976515 | + | BFO_RS03910 | DUF4974 domain-containing protein |
| 976641 | 979229 | - | BFO_RS03915 | hypothetical protein |
| **PP_island_7** | | | | |
| 1278139 | 1278321 | + | BFO_RS05160 | hypothetical protein |
| 1278355 | 1279743 | + | BFO_RS05165 | hypothetical protein |
| 1280039 | 1283056 | + | BFO_RS05170 | TonB-dependent receptor |
| 1283068 | 1284609 | + | BFO_RS05175 | RagB/SusD family nutrient uptake outer membrane protein |
| 1284503 | 1285420 | + | BFO_RS05180 | hypothetical protein |
| 1285429 | 1286235 | + | BFO_RS05185 | phospholipase |
| 1286232 | 1287584 | + | BFO_RS05190 | hypothetical protein |
| 1287786 | 1288343 | - | BFO_RS05195 | hypothetical protein |
| **PP_island_8** | | | | |
| 1364881 | 1365090 | + | BFO_RS14910 | hypothetical protein |
| 1365110 | 1365298 | + | BFO_RS05565 | hypothetical protein |
| 1365547 | 1366704 | + | BFO_RS05570 | auxiliary transporter membrane fusion protein |
| 1366719 | 1368878 | + | BFO_RS05575 | peptidase domain-containing ABC transporter |
| 1368875 | 1370182 | + | BFO_RS05580 | TolC family protein |
| 1370160 | 1370543 | + | BFO_RS14400 | hypothetical protein |
| 1370941 | 1371417 | + | BFO_RS05590 | hypothetical protein |
| **PP_island_9** | | | | |
| 1372011 | 1372373 | + | BFO_RS05600 | hypothetical protein |
| 1373398 | 1376028 | + | BFO_RS05605 | DUF5117 domain-containing protein |
| 1376065 | 1379400 | + | BFO_RS05610 | SusC/RagA family TonB-linked outer membrane protein |
| 1379414 | 1380763 | + | BFO_RS05615 | RagB/SusD family nutrient uptake outer membrane protein |
| 1380760 | 1380960 | - | BFO_RS05620 | hypothetical protein |
| 1381026 | 1381961 | - | BFO_RS05625 | IS1595 family transposase |
| 1382094 | 1384112 | - | BFO_RS05630 | membrane protein |
| **PP_island_10** | | | | |
| 1624888 | 1625613 | - | BFO_RS06610 | nitroreductase family protein |
| 1626801 | 1628309 | - | BFO_RS06620 | SusD/RagB family nutrient-binding outer membrane lipoprotein |
| 1628321 | 1631704 | - | BFO_RS06625 | SusC/RagA family TonB-linked outer membrane protein |
| 1631735 | 1632751 | - | BFO_RS06630 | DUF4974 domain-containing protein |
| 1632874 | 1633449 | + | BFO_RS06635 | RNA polymerase sigma-70 factor |
| 1633444 | 1634193 | - | BFO_RS06640 | nucleotidyltransferase family protein |
| 1634190 | 1635623 | - | BFO_RS06645 | phosphotransferase |
| **PP_island_11** | | | | |
| 1717992 | 1718813 | + | BFO_RS07040 | zinc transporter ZupT |
| 1718931 | 1719506 | + | BFO_RS07045 | SRPBCC family protein |
| 1719540 | 1720328 | + | BFO_RS07050 | class I SAM-dependent methyltransferase |
| 1720344 | 1720964 | + | BFO_RS07055 | class I SAM-dependent methyltransferase |
| 1721043 | 1721696 | + | BFO_RS07060 | class I SAM-dependent methyltransferase |
| 1721775 | 1722590 | + | BFO_RS07065 | hypothetical protein |
| 1722708 | 1723454 | + | BFO_RS07070 | class I SAM-dependent methyltransferase |
| 1723504 | 1724076 | + | BFO_RS07075 | hypothetical protein |
| 1724073 | 1724468 | + | BFO_RS07080 | glyoxalase/bleomycin resistance/dioxygenase family protein |
| 1724790 | 1726541 | + | BFO_RS07085 | ABC transporter ATP-binding protein |
| 1726618 | 1726983 | - | BFO_RS07090 | four helix bundle protein |
| 1727071 | 1728801 | + | BFO_RS07095 | ABC transporter ATP-binding protein |
| 1728873 | 1729799 | + | BFO_RS07100 | glutaminase |
| **PP_island_12** | | | | |
| 1863319 | 1863507 | + | BFO_RS07615 | hypothetical protein |
| 1863562 | 1863753 | + | BFO_RS07620 | hypothetical protein |
| 1863750 | 1864745 | + | BFO_RS07625 | DUF288 domain-containing protein, partial |
| 1865169 | 1866170 | + | BFO_RS07635 | hypothetical protein |
| 1866342 | 1867550 | - | BFO_RS07640 | ROK family transcriptional regulator |
| 1867793 | 1868146 | - | BFO_RS07645 | MarR family transcriptional regulator |
| 1868164 | 1868622 | - | BFO_RS07650 | thioredoxin |
| 1868631 | 1868831 | - | BFO_RS14990 | hypothetical protein |
| 1868847 | 1871291 | - | BFO_RS07655 | pyridine nucleotide-disulfide oxidoreductase |
| **PP_island_13** | | | | |
| 2194699 | 2195094 | - | BFO_RS08925 | hypothetical protein |
| 2195130 | 2195336 | - | BFO_RS08930 | hypothetical protein |
| 2195468 | 2196565 | + | BFO_RS08935 | histidine kinase |
| 2196584 | 2197333 | + | BFO_RS08940 | DNA-binding response regulator |
| 2197770 | 2197892 | + | BFO_RS15040 | MULTISPECIES: glycine cleavage system H protein |
| 2198059 | 2198994 | - | BFO_RS08945 | IS1595 family transposase |
| 2199294 | 2199512 | + | BFO_RS15045 | hypothetical protein |
| 2199524 | 2199805 | + | BFO_RS08955 | hypothetical protein |
| **PP_island_14** | | | | |
| 2249858 | 2250733 | + | BFO_RS09165 | ABC transporter ATP-binding protein |
| 2250740 | 2251414 | + | BFO_RS09170 | hypothetical protein |
| 2251438 | 2252661 | + | BFO_RS09175 | DUF4857 domain-containing protein |
| 2252690 | 2253235 | + | BFO_RS09180 | hypothetical protein |
| 2253238 | 2253807 | + | BFO_RS09185 | peptidase |
| 2253959 | 2254432 | - | BFO_RS09190 | hypothetical protein |
| 2254460 | 2255356 | - | BFO_RS09195 | hypothetical protein |
| 2255756 | 2256946 | - | BFO_RS09200 | hypothetical protein |
| **PP_island_15** | | | | |
| 2351164 | 2352420 | + | BFO_RS09565 | hypothetical protein |
| 2352547 | 2352984 | + | BFO_RS09570 | hypothetical protein |
| 2354089 | 2355003 | + | BFO_RS09580 | N-acetylneuraminate lyase |
| 2355009 | 2356187 | + | BFO_RS09585 | N-acylglucosamine 2-epimerase |
| 2356212 | 2357456 | + | BFO_RS09590 | MFS transporter |
| 2357489 | 2360824 | + | BFO_RS09595 | TonB-dependent receptor |
| 2360849 | 2362420 | + | BFO_RS09600 | SusD family outer membrane lipoprotein NanU |
| 2362503 | 2364122 | + | BFO_RS09605 | sialidase |
| 2364119 | 2366116 | + | BFO_RS09610 | beta-N-acetylhexosaminidase |
| 2366134 | 2368212 | + | BFO_RS09615 | sialate O-acetylesterase |
| 2368220 | 2369434 | + | BFO_RS09620 | cyclically-permuted mutarotase family protein |
| **PP_island_16** | | | | |
| 2444368 | 2444754 | - | BFO_RS09910 | diacylglycerol kinase |
| 2445130 | 2446347 | + | BFO_RS09915 | glucuronyl hydrolase |
| 2446405 | 2449530 | + | BFO_RS09920 | TonB-dependent receptor |
| 2449542 | 2451248 | + | BFO_RS09925 | RagB/SusD family nutrient uptake outer membrane protein |
| 2451447 | 2452289 | + | BFO_RS09930 | 5-dehydro-4-deoxy-D-glucuronate isomerase |
| 2452303 | 2453445 | + | BFO_RS09935 | DUF4861 domain-containing protein |
| 2453442 | 2454992 | + | BFO_RS09940 | arylsulfatase |
| 2455029 | 2456954 | + | BFO_RS09945 | heparinase |
| 2456989 | 2458029 | + | BFO_RS09950 | sugar kinase |
| 2458066 | 2458857 | + | BFO_RS09955 | 3-oxoacyl-ACP reductase |
| 2458884 | 2459555 | + | BFO_RS09960 | bifunctional 4-hydroxy-2-oxoglutarate aldolase/2-dehydro-3-deoxy-phosphogluconate aldolase |
| 2459605 | 2461032 | + | BFO_RS09965 | MFS transporter |
| **PP_island_17** | | | | |
| 2490319 | 2490903 | + | BFO_RS10105 | RNA polymerase sigma-70 factor |
| 2491036 | 2491836 | + | BFO_RS10110 | CRISPR-associated endoribonuclease Cas6 |
| 2491862 | 2492485 | + | BFO_RS10115 | plasmid pRiA4b ORF-3 family protein |
| 2492553 | 2493278 | + | BFO_RS10120 | type I-PGING CRISPR-associated protein Cas5p |
| 2493271 | 2494812 | + | BFO_RS10125 | type I-PGING CRISPR-associated protein Cas8c/Csp2 |
| 2494832 | 2495749 | + | BFO_RS10130 | type I-PGING CRISPR-associated protein Cas7/Csp1 |
| 2495826 | 2498021 | + | BFO_RS10135 | CRISPR-associated helicase Cas3\' |
| **PP_island_18** | | | | |
| 2826845 | 2827807 | - | BFO_RS11520 | acetylornithine carbamoyltransferase |
| 2828032 | 2828979 | + | BFO_RS11525 | DUF4974 domain-containing protein |
| 2829102 | 2832320 | + | BFO_RS11530 | SusC/RagA family TonB-linked outer membrane protein |
| 2832332 | 2834077 | + | BFO_RS11535 | RagB/SusD family nutrient uptake outer membrane protein |
| 2834126 | 2835607 | + | BFO_RS11540 | beta-glycosidase |
| 2836111 | 2836845 | + | BFO_RS11545 | NUDIX hydrolase |
| 2836899 | 2838395 | + | BFO_RS11550 | carbohydrate kinase |
| 2838435 | 2839751 | + | BFO_RS11555 | xylose isomerase |
| 2839791 | 2841290 | + | BFO_RS11560 | D-xylose transporter XylE |
| 2841426 | 2841617 | + | BFO_RS11565 | hypothetical protein |
| 2842358 | 2845822 | + | BFO_RS11580 | SusC/RagA family TonB-linked outer membrane protein |
| 2845835 | 2847481 | + | BFO_RS11585 | RagB/SusD family nutrient uptake outer membrane protein |
| 2847472 | 2849127 | + | BFO_RS11590 | DUF5018 domain-containing protein |
| 2849160 | 2850575 | + | BFO_RS11595 | S-layer protein |
| 2850630 | 2852090 | + | BFO_RS11600 | Ser/Thr phosphatase |
