# Supplementary figures and images for: Comparative genome characterization of the periodontal pathogen Tannerella forsythia
Source: BMC Genomics. 2020 Feb 11;21:150. doi: 10.1186/s12864-020-6535-y (PMC7014623; doi:10.1186/s12864-020-6535-y)

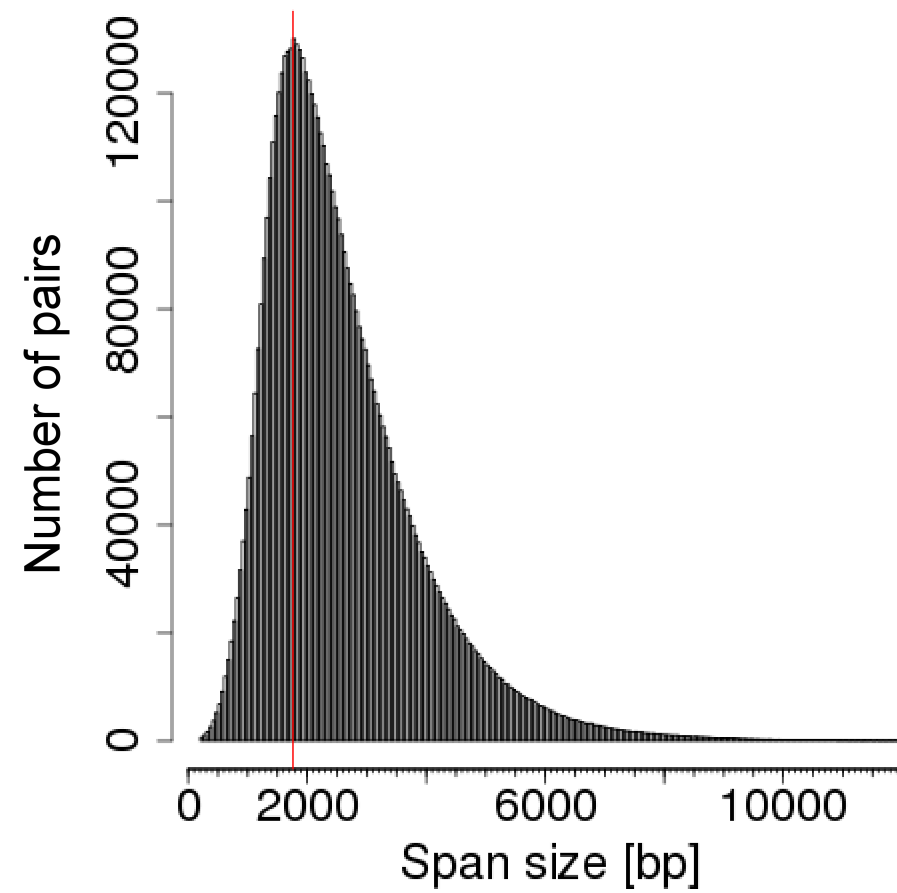

Supplement: Supplementary file 10 — Additional file 10: Figure S1. Span size distribution of the mate-pair library prepared from DNA of T. forsythia strain ATCC 43037. The peak of the distribution is at 1759 bp, indicated by the red line. [file 12864_2020_6535_MOESM10_ESM.pdf]

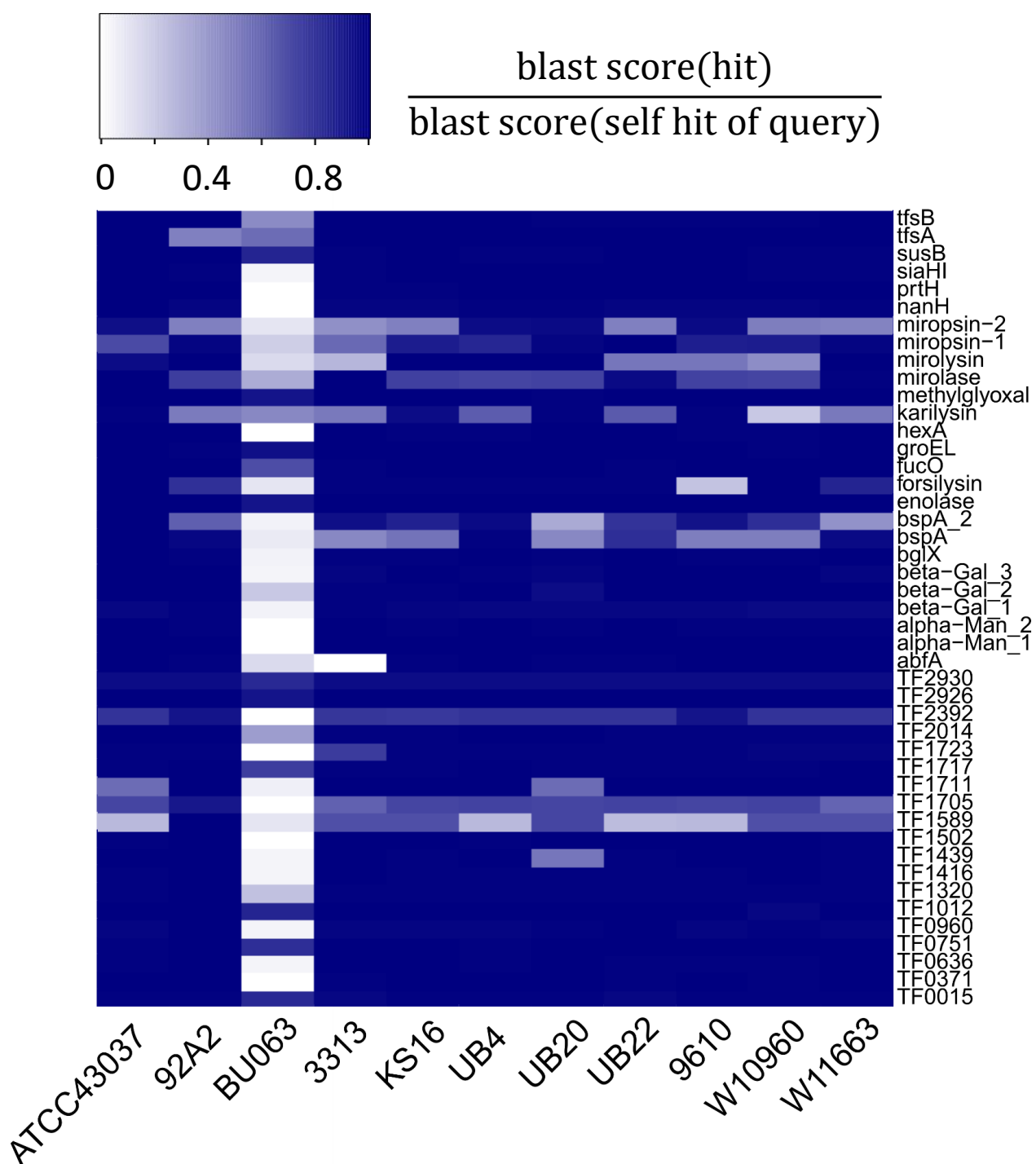

Supplement: Supplementary file 11 — Additional file 11: Figure S2. Blast Score Ratio values plotted as heatmap for 45 suggested virulence genes in ten T. forsythia strains and the genome of a putative health-associated Tannerella sp. BU063. In contrast to Fig. 4, the 45 gene sequences were blasted against sequences of annotated CDS in each genome. [file 12864_2020_6535_MOESM11_ESM.pdf]
