# Supplementary material for: Comparative genome characterization of the periodontal pathogen Tannerella forsythia
Source: BMC Genomics. 2020 Feb 11;21:150. doi: 10.1186/s12864-020-6535-y (PMC7014623; doi:10.1186/s12864-020-6535-y)
Supplement: Supplementary file 12 — Additional file 12: File S1. Alignments of the sequence KP715369 to T. forsythia genomes. [file 12864_2020_6535_MOESM12_ESM.doc]

**Alignments of KP715369 to *T. forsythia* genomes**

KP715369 aligns partly to scaffold 1 and partly to scaffold 2 of the new *T. forsythia* ATCC 43037 assembly:

| Query Start | Query End | Query Frame | Subject Start | Subject End | Subject Frame | Alignment length | Bit Score | E-Vaule |
| --- | --- | --- | --- | --- | --- | --- | --- | --- |
| 1 | 7,196 | 1 | 1,316,685 | 1,323,879 | -1 | 7,197 | 13132.6 | 0 |
| 6,740 | 15,010 | 1 | 518,902 | 527,168 | -1 | 8,274 | 14971.9 | 0 |

These two alignments are displayed in the darker color in Figures 1-4 below. Note that whereas the query regions covered by these alignments slightly overlap, this is not directly visible in Figures 1-4, as smaller alignments are displayed as well.

In the three available complete *T. forsythia* genomes alignments of KP715369 can mostly be found in single regions. Alignments in other regions are also produced by KP715369 (Figures 5-8).

Alignments were obtained with blastn [1, 2] using default parameters.

Visualizations provided in this document were generated using Kablammo [3].

References:

1. Zhang Z, Schwartz S, Wagner L, Miller W. A Greedy Algorithm for Aligning DNA Sequences. J Comput Biol. 2000;7:203-14.

2. Camacho C, Coulouris G, Avagyan V, Ma N, Papadopoulos J, Bealer K, et al. BLAST+: architecture and applications. BMC Bioinformatics. 2009;10:421.

3. Wintersinger JA, Wasmuth JD. Kablammo: an interactive, web-based BLAST results visualizer. Bioinformatics. 2015;31:1305-6.


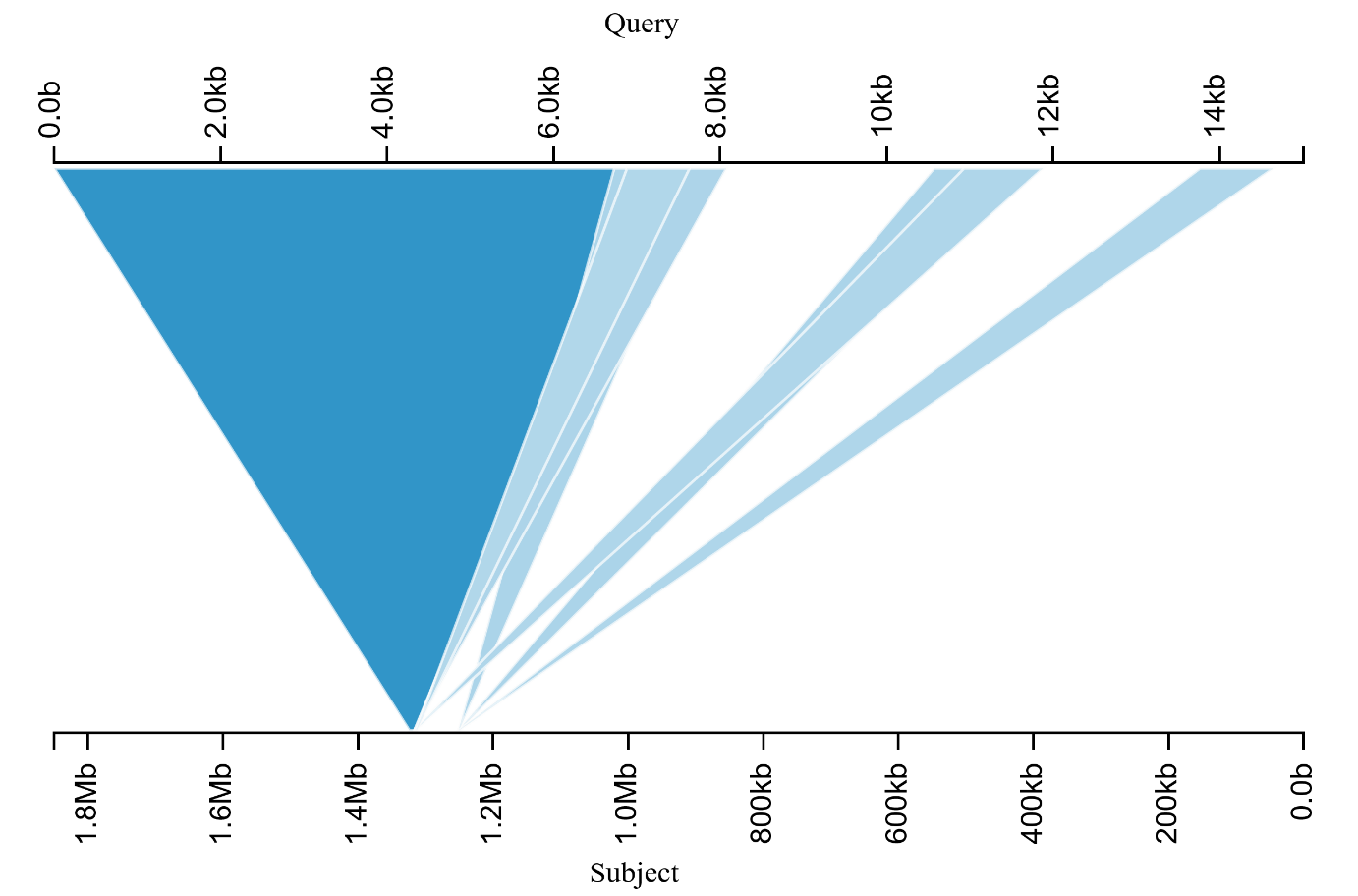


Figure 1: Fragment of KP715369 aligning to ATCC 43037 scaffold 1
E-value cut-off 10^-5^, alignments of size > 5% of query length shown


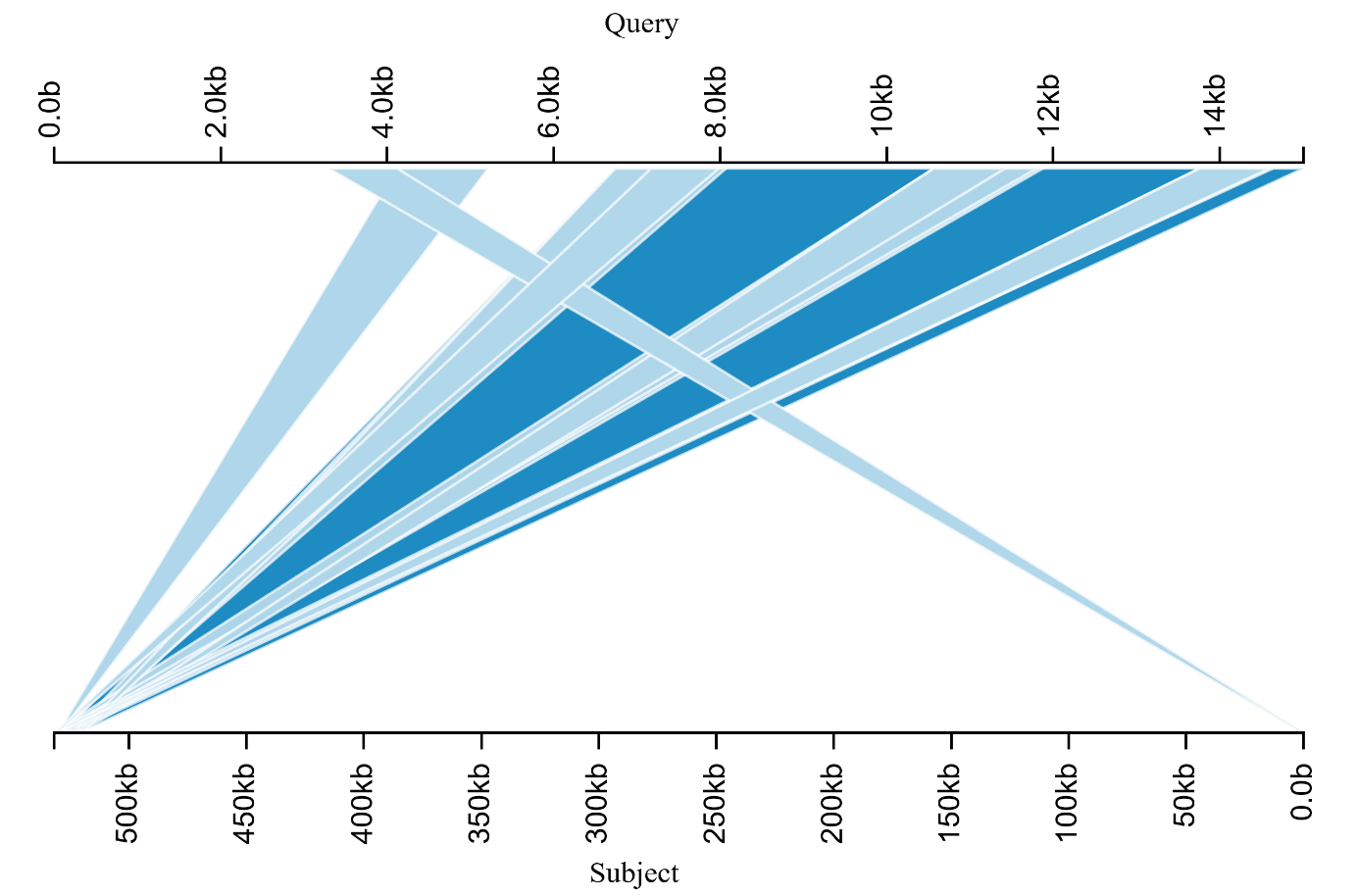


Figure 2: Fragment of KP715369 aligning to ATCC 43037 scaffold 2
E-value cut-off 10^-5^, alignments of size > 5% of query length shown


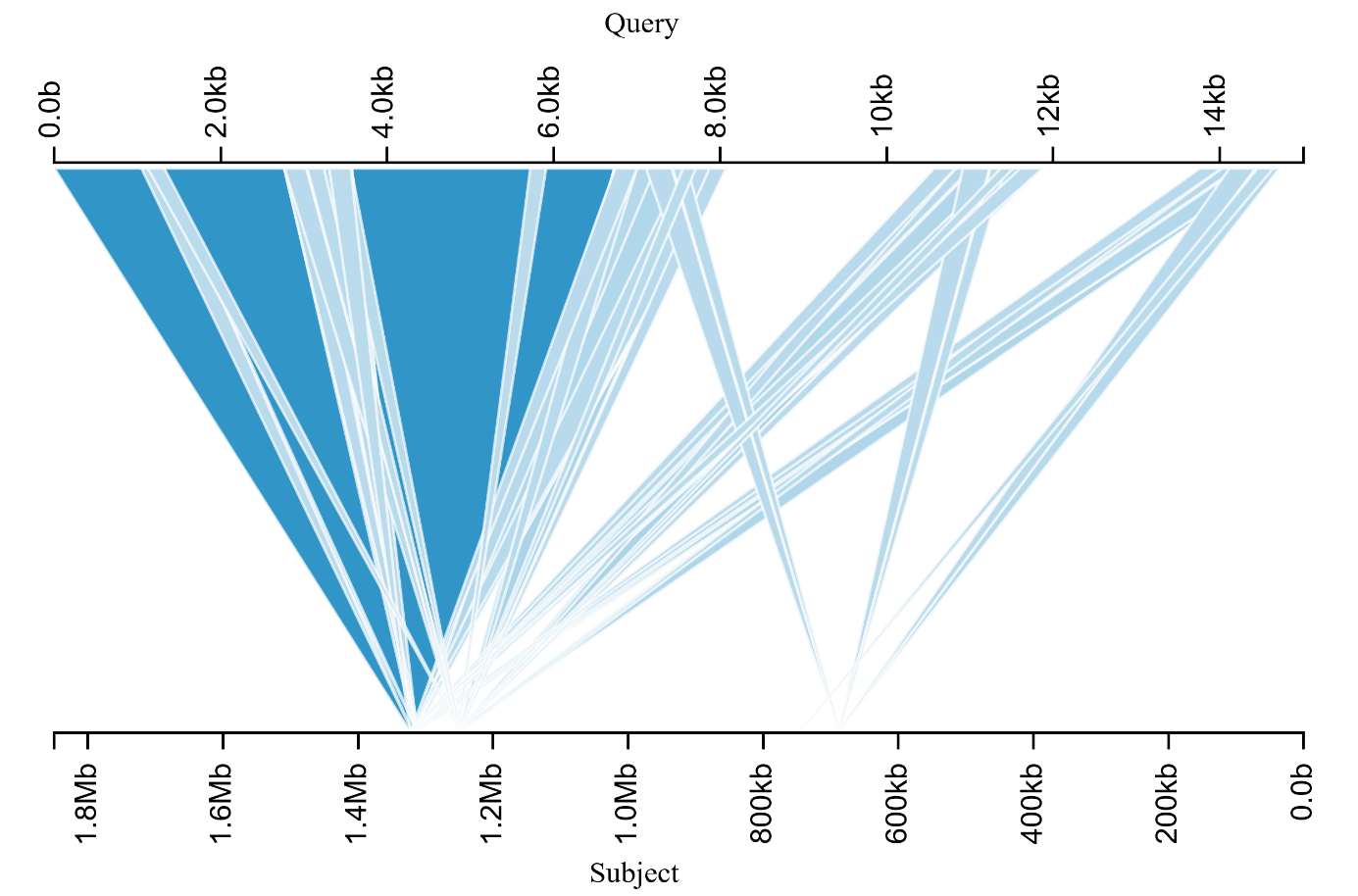


Figure 3: Fragment of KP715369 aligning to ATCC 43037 scaffold 1
E-value cut-off 10^-5^, alignments of size > 1% of query length shown


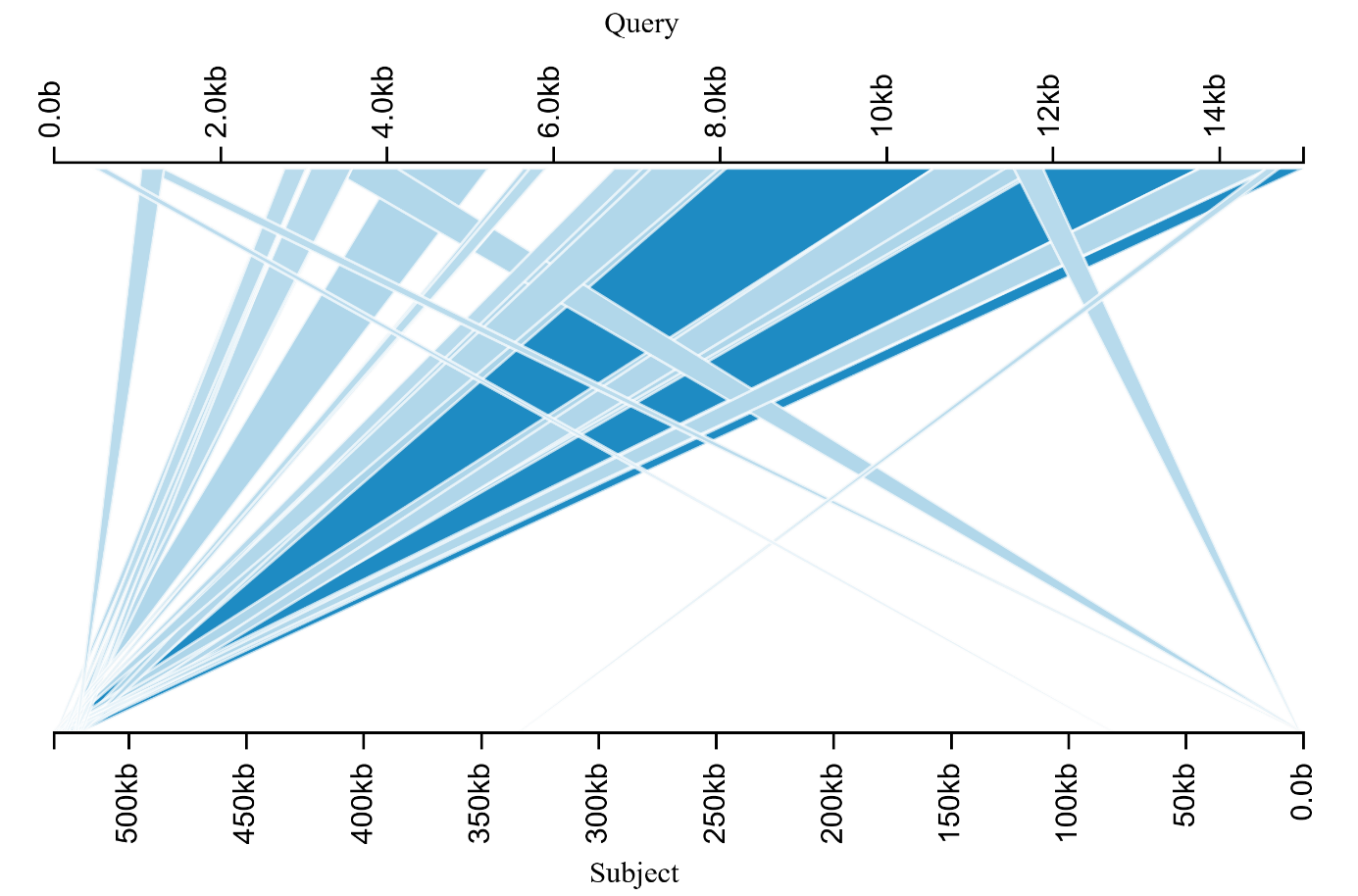


Figure 4: Fragment of KP715369 aligning to ATCC 43037 scaffold 2
E-value cut-off 10^-5^, alignments of size > 1% of query length shown


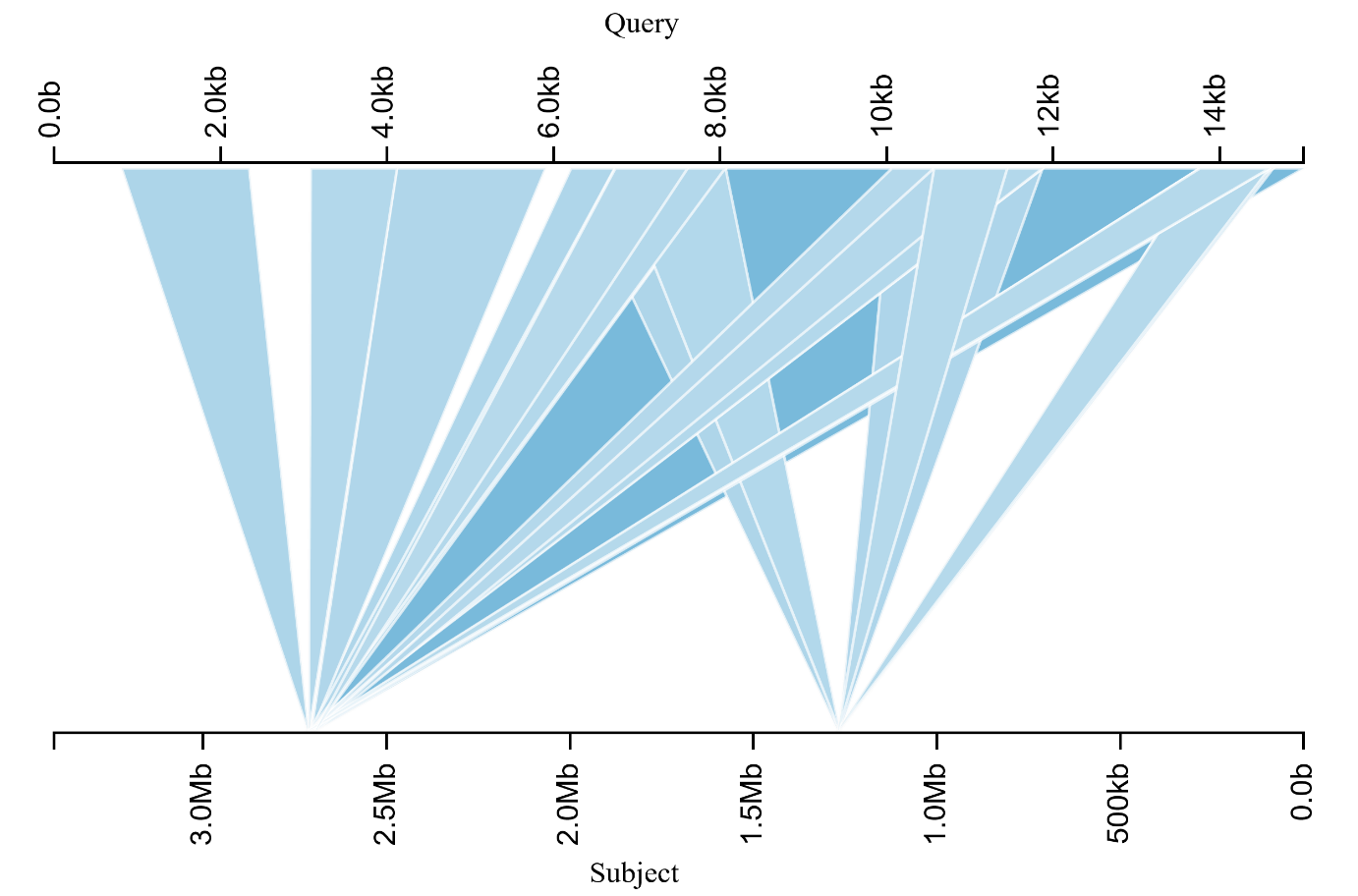


Figure 5: KP715369 vs FDC 92A
E-value cut-off 10^-5^, alignments of size > 5% of query length shown


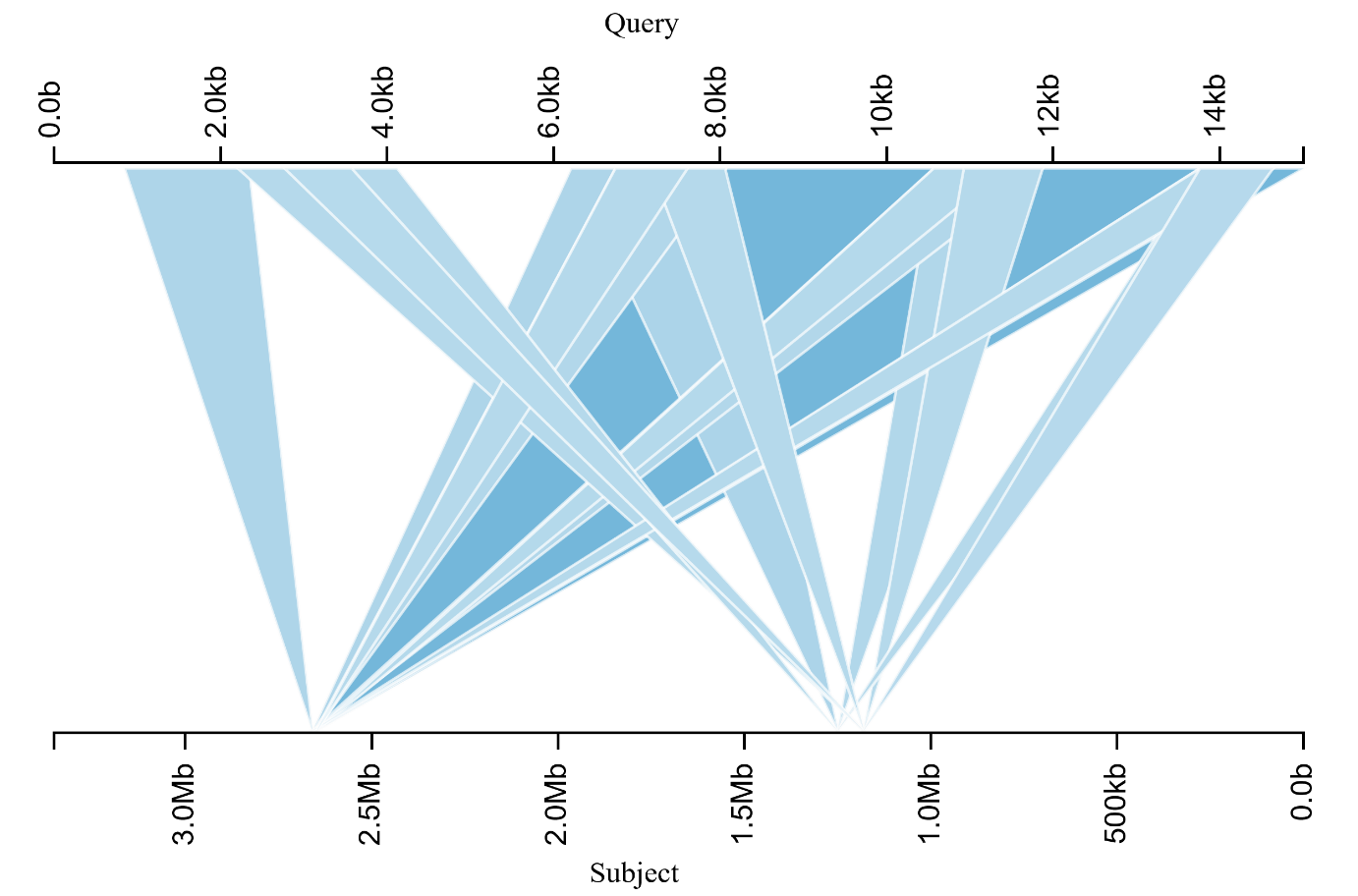


Figure 6: KP715369 vs 3313
E-value cut-off 10^-5^, alignments of size > 5% of query length shown


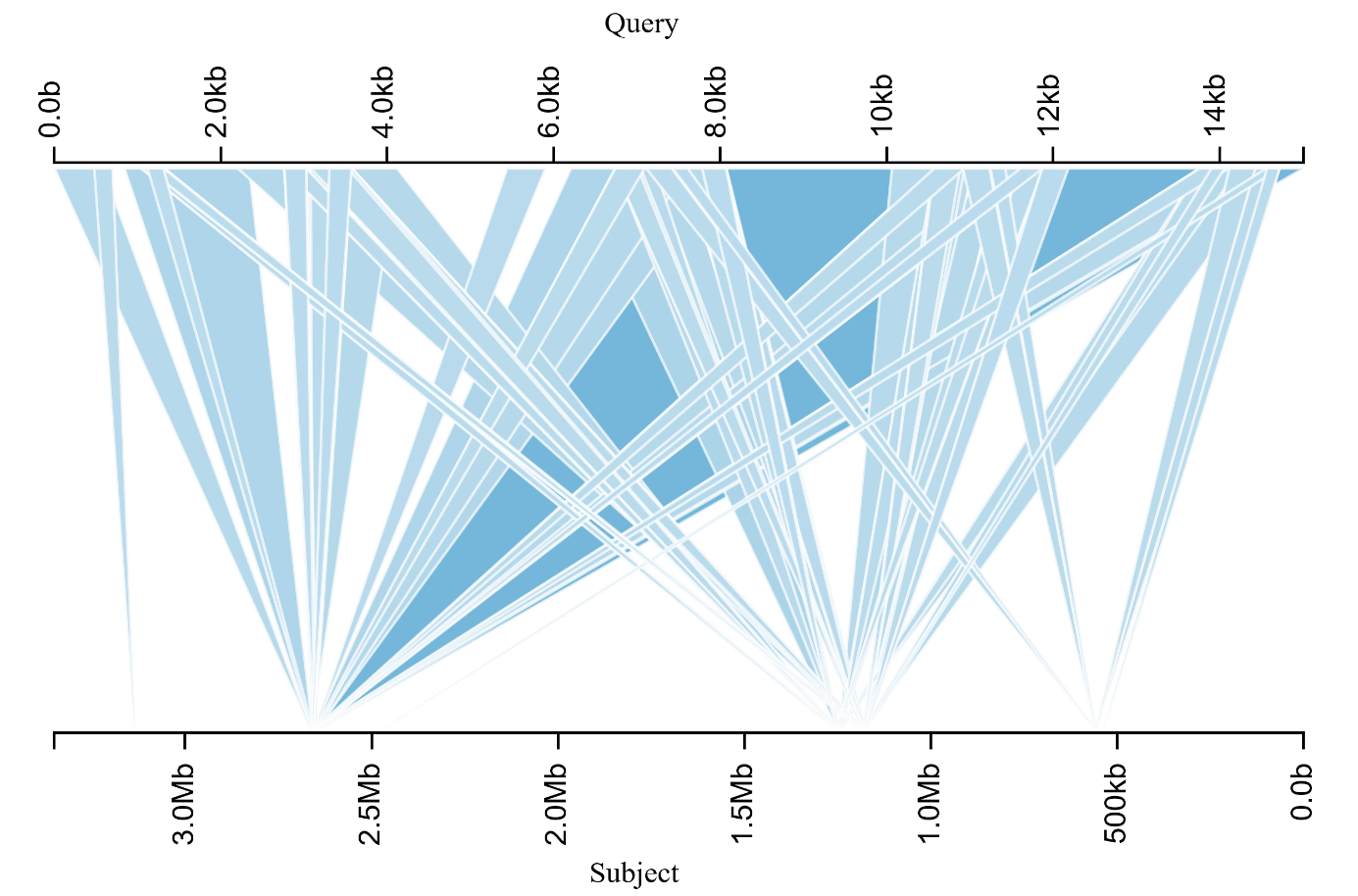


Figure 7: KP715369 vs 3313
E-value cut-off 10^-5^, alignments of size > 1% of query length shown


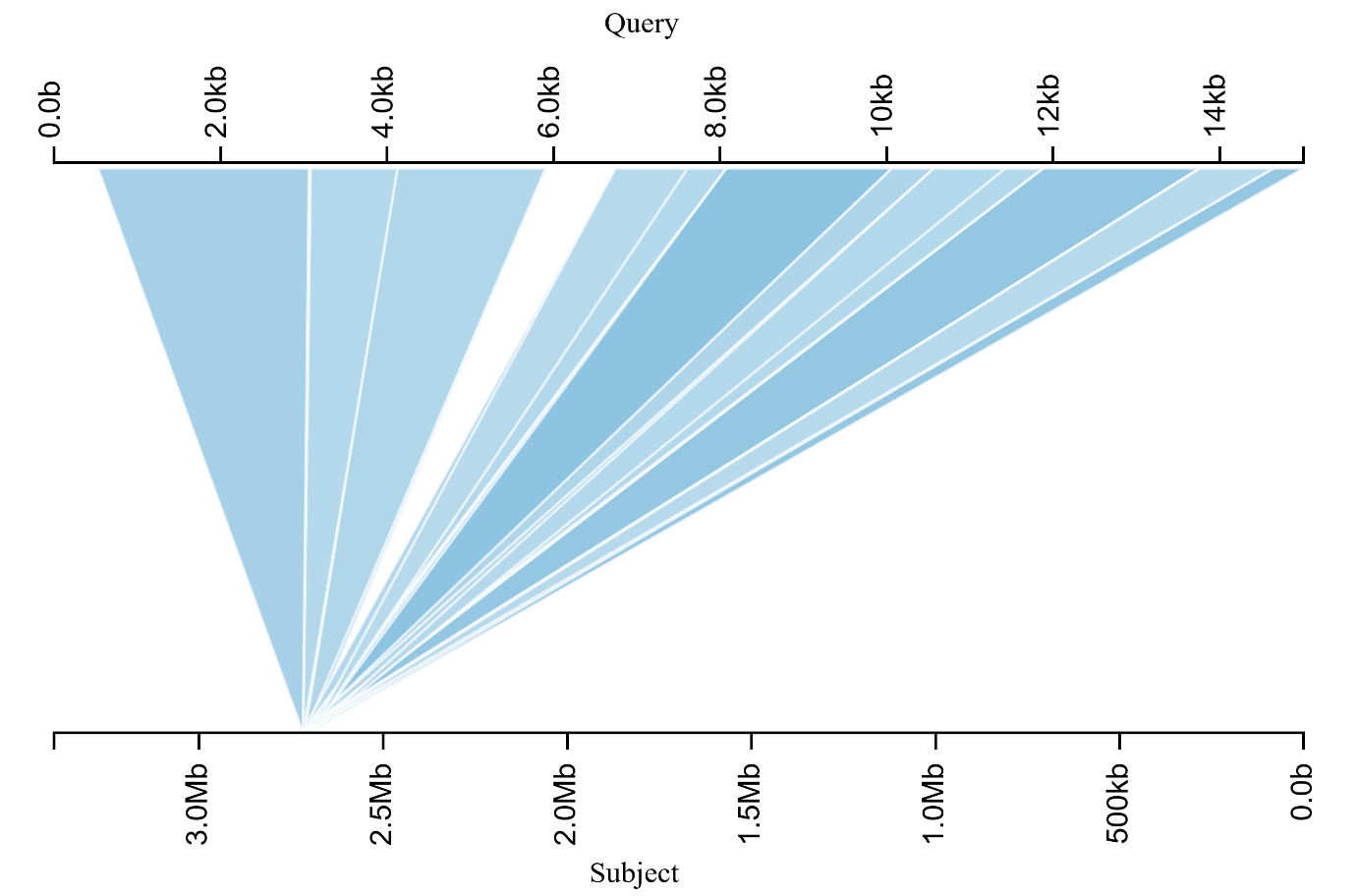


Figure 8: KP715369 vs KS16
E-value cut-off 10^-5^, alignments of size > 5% of query length shown
