# Supplementary material for: Comparative genome characterization of the periodontal pathogen Tannerella forsythia
Source: BMC Genomics. 2020 Feb 11;21:150. doi: 10.1186/s12864-020-6535-y (PMC7014623; doi:10.1186/s12864-020-6535-y)
Supplement: Supplementary file 13 — Additional file 13: File S2. Detailed view on the alignment of T. forsythia karilysin to a putative orthologue in Tannerella sp. BU063. [file 12864_2020_6535_MOESM13_ESM.doc]

**Karilysin in *Tannerella* sp. BU063**

Alignment of *T. forsythia* ATCC 43037 Karilysin amino acid sequence (top) to a putative orthologue i*n Tannerella* sp. BU063 (WP_083206853.1).

Query coverage: 97.5%

Sequence identity: 53.1%


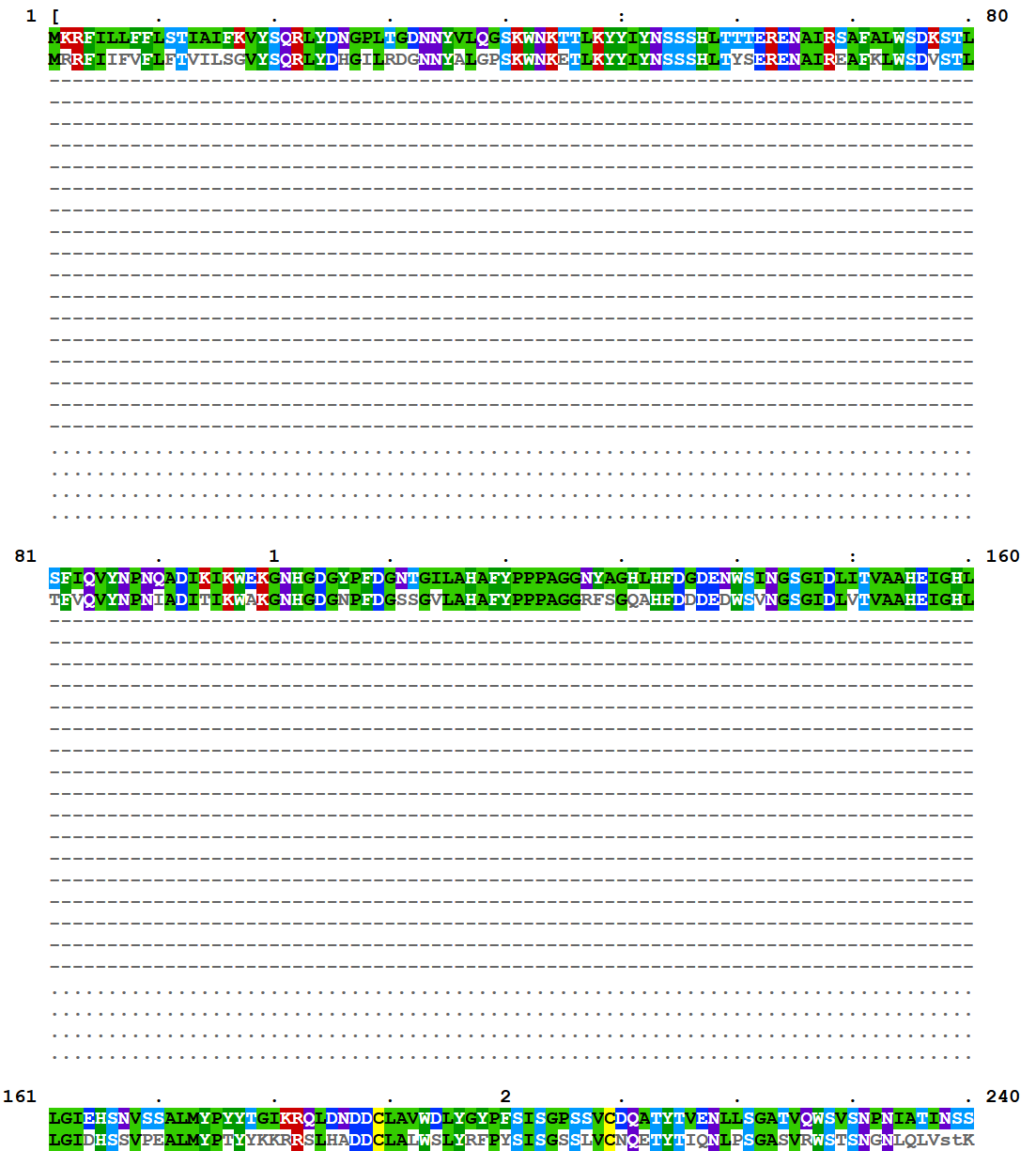

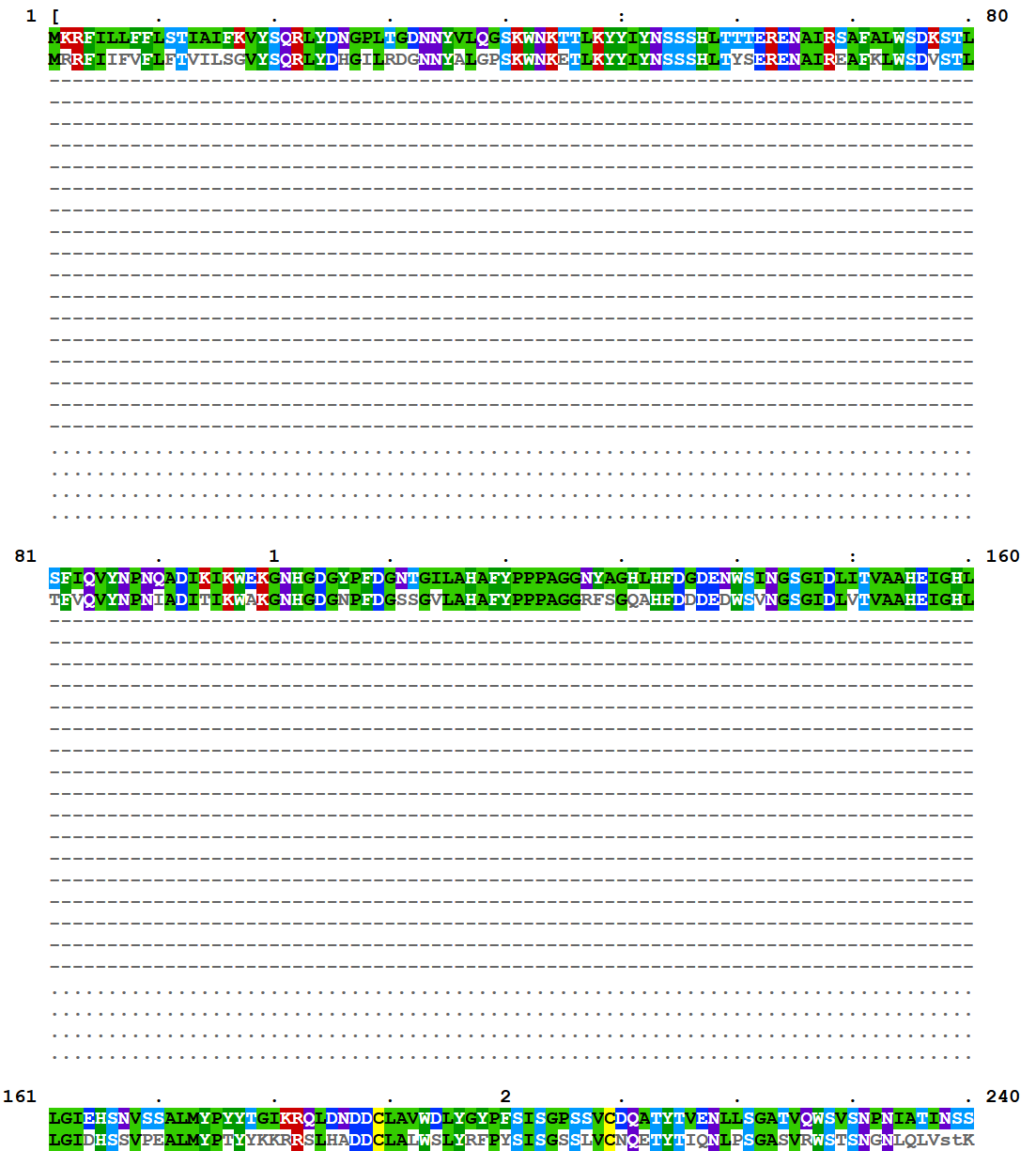

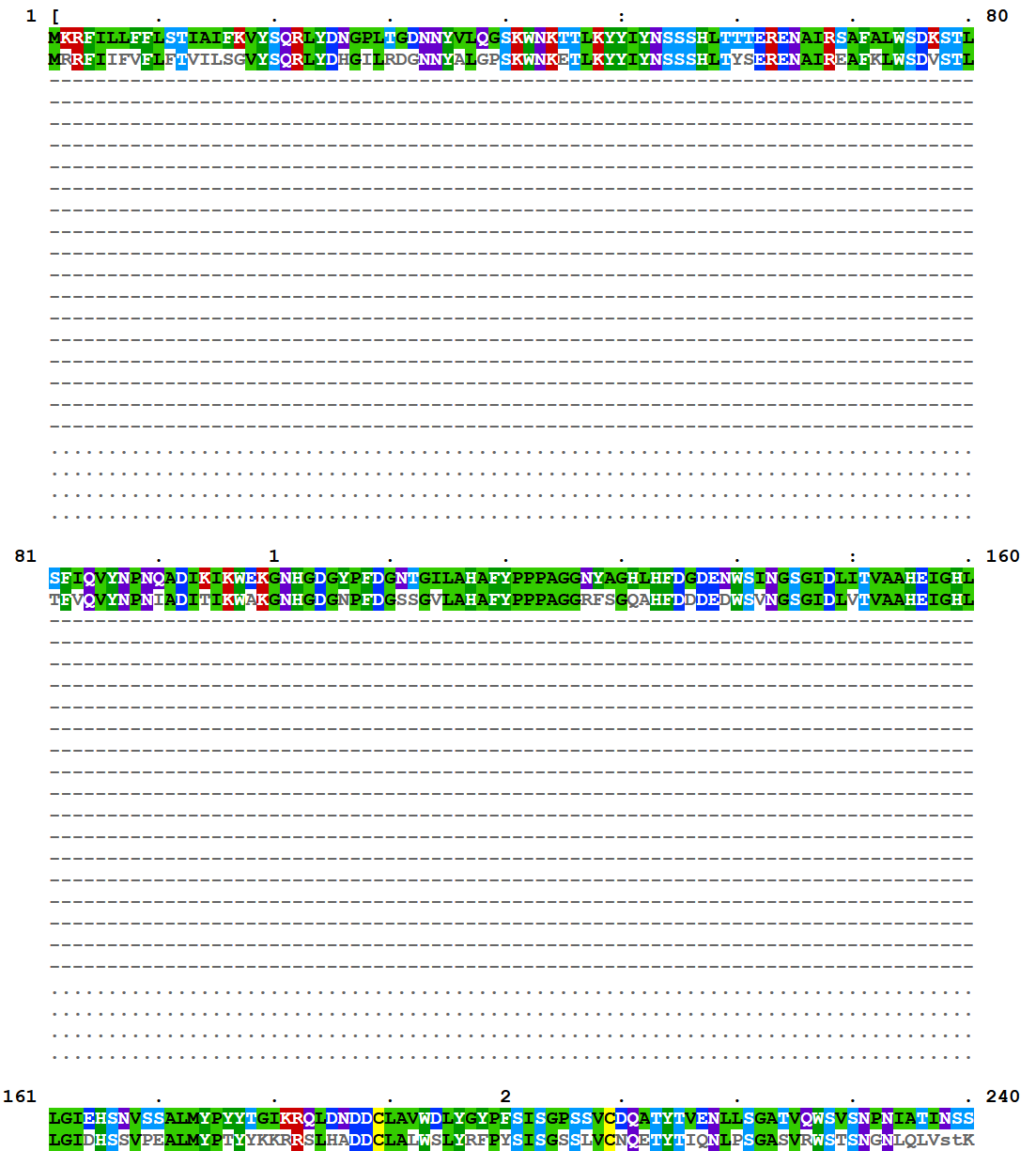

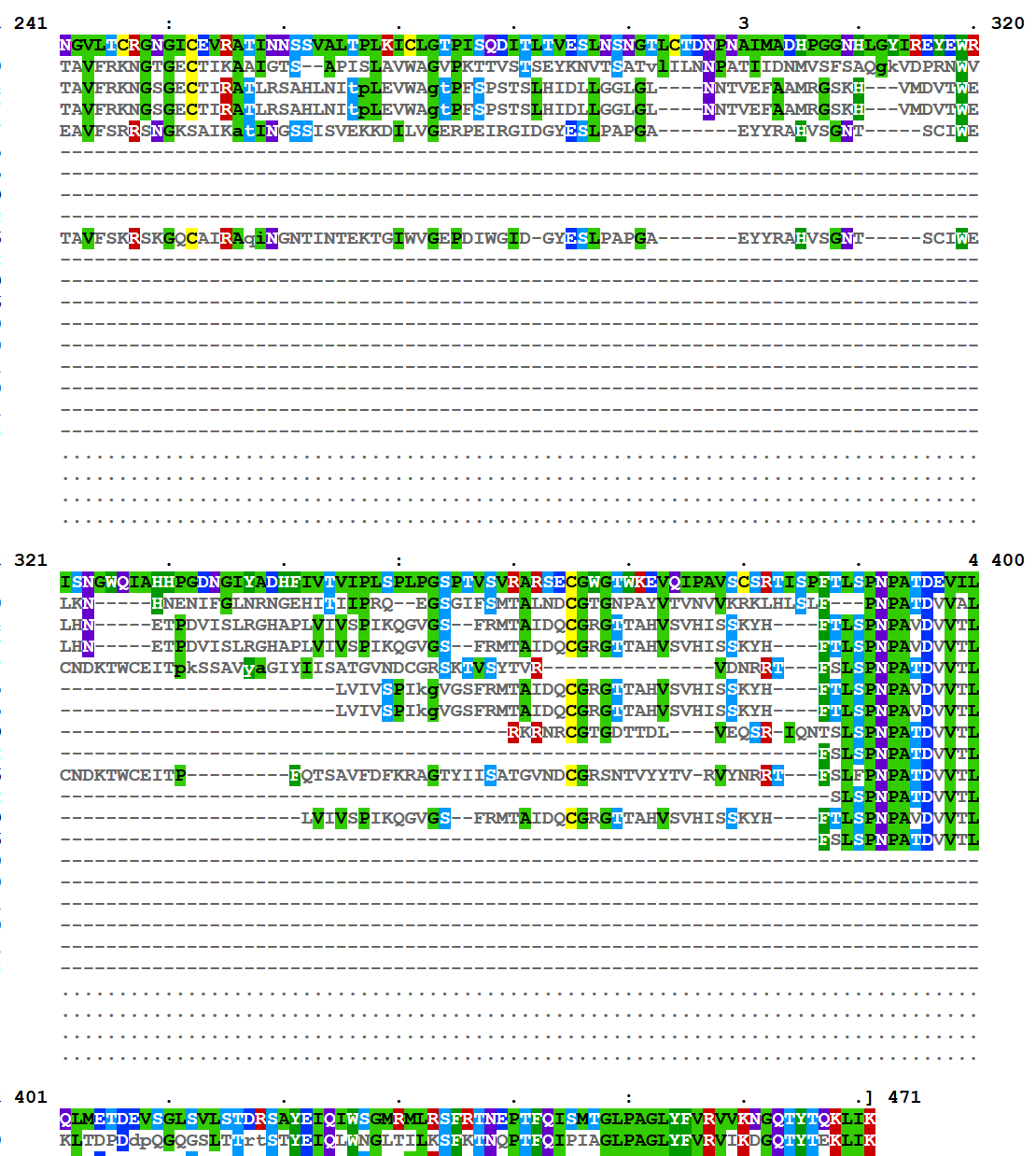

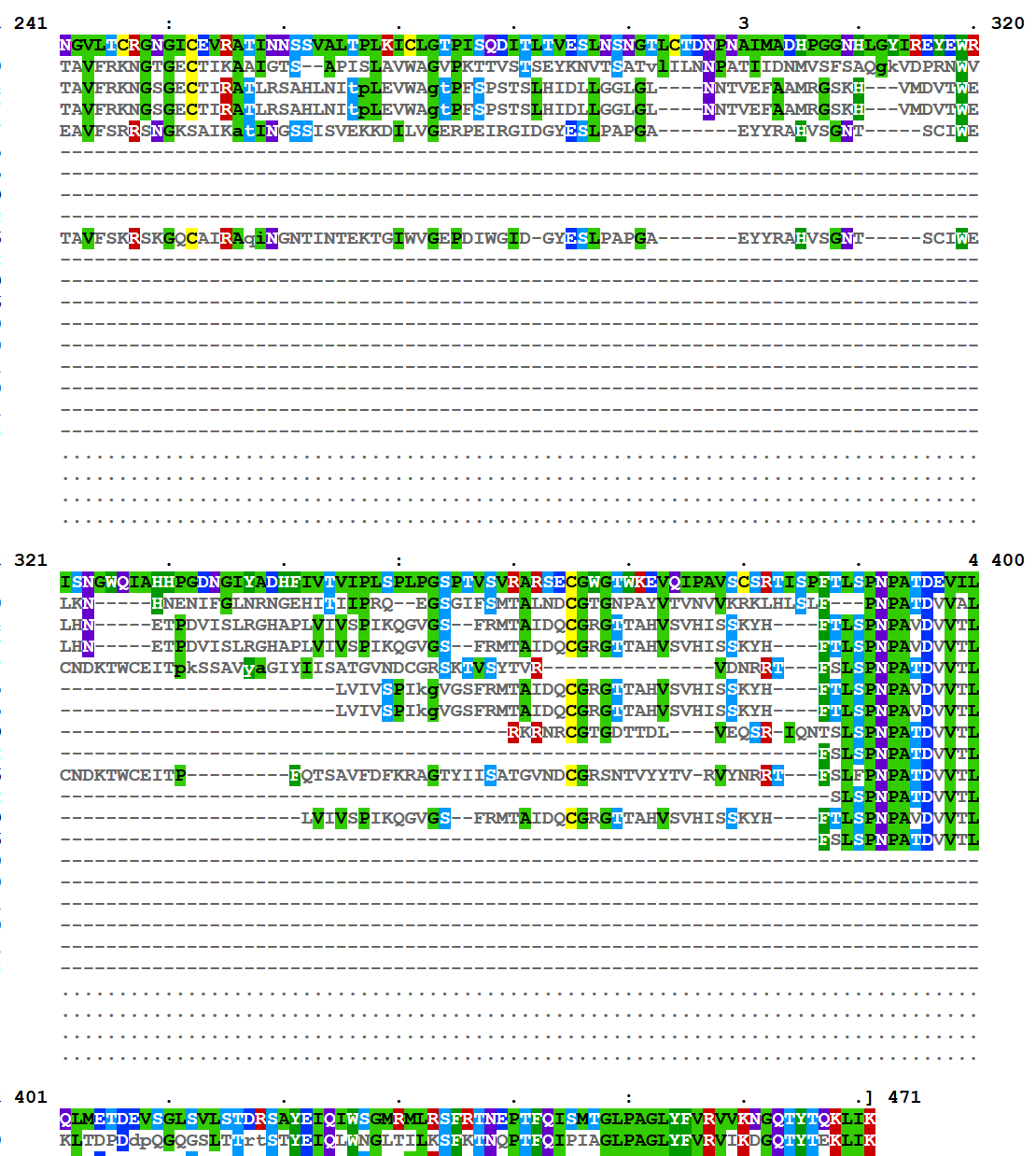

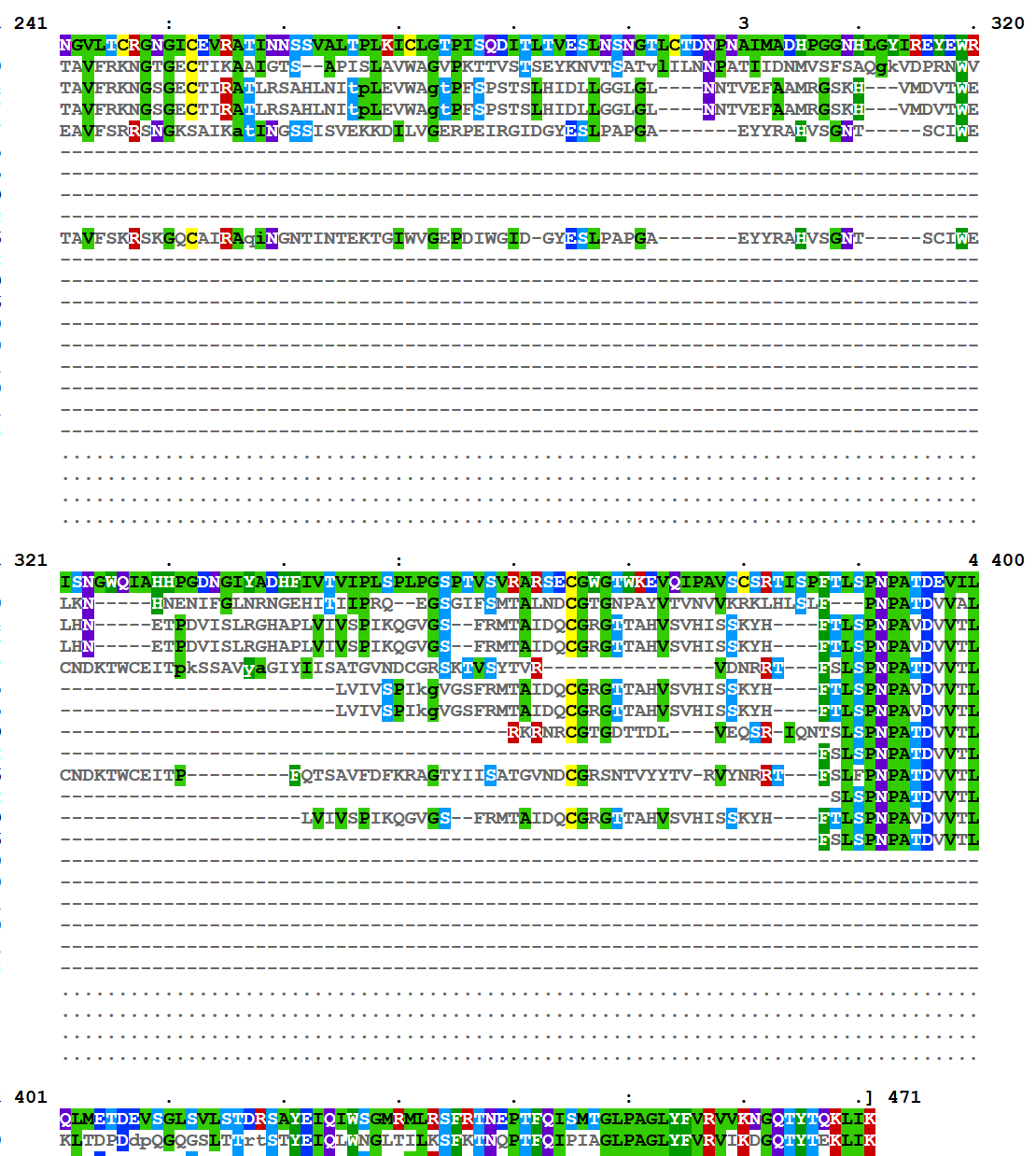


Conserved domains in the query sequence according to the Conserved Domain Database (CDD) [1-4]:


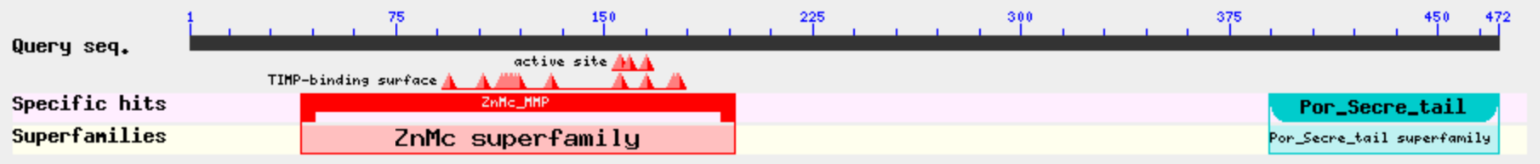


ZnMc_MMP cd04278
Zinc-dependent metalloprotease, matrix metalloproteinase (MMP) sub-family. MMPs are ...
Positions: 41-197 E-Value: 6.65e-67

Por_Secre_tail TIGR04183
Por secretion system C-terminal sorting domain; Species that include Porphyromonas gingivalis, ...
Positions: 389-472 E-Value: 5.85e-08

The alignment was generated with blastp [5,6] and visualized with MView [7-9].

References:

1. Marchler-Bauer A, Bo Y, Han L, He J, Lanczycki CJ, Lu S, et al. CDD/SPARCLE: functional classification of proteins via subfamily domain architectures. Nucleic Acids Res. 2017;45:D200-3.

2. Marchler-Bauer A, Derbyshire MK, Gonzales NR, Lu S, Chitsaz F, Geer LY, et al. CDD: NCBI’s conserved domain database. Nucleic Acids Res. 2015;43:D222–6.

3. Marchler-Bauer A, Lu S, Anderson JB, Chitsaz F, Derbyshire MK, DeWeese-Scott C, et al. CDD: a Conserved Domain Database for the functional annotation of proteins. Nucleic Acids Res. 2011;39 Database issue:D225-229.

4. Marchler-Bauer A, Bryant SH. CD-Search: protein domain annotations on the fly. Nucleic Acids Res. 2004;32 Web Server:W327–31.

5. Altschul SF, Madden TL, Schäffer AA, Zhang J, Zhang Z, Miller W, et al. Gapped BLAST and PSI-BLAST: a new generation of protein database search programs. Nucleic Acids Res. 1997;25:3389–402.

6. Camacho C, Coulouris G, Avagyan V, Ma N, Papadopoulos J, Bealer K, et al. BLAST+: architecture and applications. BMC Bioinformatics. 2009;10:421.

7. Brown NP, Leroy C, Sander C. MView: a web-compatible database search or multiple alignment viewer. Bioinforma Oxf Engl. 1998;14:380–1.

8. Li W, Cowley A, Uludag M, Gur T, McWilliam H, Squizzato S, et al. The EMBL-EBI bioinformatics web and programmatic tools framework. Nucleic Acids Res. 2015;43:W580-584.

9. McWilliam H, Li W, Uludag M, Squizzato S, Park YM, Buso N, et al. Analysis Tool Web Services from the EMBL-EBI. Nucleic Acids Res. 2013;41 Web Server issue:W597-600.
